# Small Molecule Arranged Thermal Proximity Coaggregation (smarTPCA)—A Novel Approach to Characterize Protein–Protein Interactions in Living Cells by Similar Isothermal Dose–Responses

**DOI:** 10.3390/ijms23105605

**Published:** 2022-05-17

**Authors:** Thomas Lenz, Kai Stühler

**Affiliations:** 1 Molecular Proteomics Laboratory, Biological Medical Research Center, Heinrich-Heine-University Düsseldorf, 40225 Düsseldorf, Germany; 2 Institute for Molecular Medicine, Proteome Research, University Hospital and Medical Faculty, Heinrich-Heine-University Düsseldorf, 40225 Düsseldorf, Germany

**Keywords:** cellular thermal shift assay (CETSA), thermal proteome profiling (TPP), isothermal dose–response CETSA (ITDR-CETSA), TPP with compound concentration range (TPP-CCR), intracellular protein–protein interaction (PPI), kinase inhibitors, thermal protein stabilization, thermal shift, small molecule arranged thermal proximity coaggregation (smarTPCA)

## Abstract

Chemical biology and the application of small molecules has proven to be a potent perturbation strategy, especially for the functional elucidation of proteins, their networks, and regulators. In recent years, the cellular thermal shift assay (CETSA) and its proteome-wide extension, thermal proteome profiling (TPP), have proven to be effective tools for identifying interactions of small molecules with their target proteins, as well as off-targets in living cells. Here, we asked the question whether isothermal dose–response (ITDR) CETSA can be exploited to characterize secondary effects downstream of the primary binding event, such as changes in post-translational modifications or protein–protein interactions (PPI). By applying ITDR-CETSA to MAPK14 kinase inhibitor treatment of living HL-60 cells, we found similar dose–responses for the direct inhibitor target and its known interaction partners MAPKAPK2 and MAPKAPK3. Extension of the dose–response similarity comparison to the proteome wide level using TPP with compound concentration range (TPP-CCR) revealed not only the known MAPK14 interaction partners MAPKAPK2 and MAPKAPK3, but also the potentially new intracellular interaction partner MYLK. We are confident that dose-dependent small molecule treatment in combination with ITDR-CETSA or TPP-CCR similarity assessment will not only allow discrimination between primary and secondary effects, but will also provide a novel method to study PPI in living cells without perturbation by protein modification, which we named “small molecule arranged thermal proximity coaggregation” (smarTPCA).

## 1. Introduction

Thermal (de)stabilization of a protein by complexation with a ligand has been known for a long time [1,2], and in the beginning it was successfully applied for routine screening of small molecule binding to recombinant purified proteins for drug discovery in so-called thermal shift assays (TSA) [3,4,5,6]. In 2013, the TSA method was extended to study small molecule binding to proteins in complex environments such as cell extracts, living cells, or tissues, and the method was accordingly named cellular thermal shift assay (CETSA) [7,8]. CETSA relies on heat-induced irreversible protein precipitation in the presence or absence of a protein-binding small molecule and on antibody-based quantitative measurements (such as immunoblotting) of the undenatured soluble protein fraction. The main strength of the CETSA method clearly lies in its ability to assess drug–protein interactions in a physiologically relevant setting within living cells. This so-called target engagement is determined by the local small molecule concentration and its binding affinity to the target protein. On one hand, the local small molecule concentration is influenced by multiple factors such as the active or passive cellular uptake (cell permeability) and cellular export, as well as its metabolism, which is summarized in pharmacokinetics as ADME (absorption, distribution, metabolism, and excretion) [9]. Whereas, on the other hand, the binding affinity to the target protein depends on its activation and interaction state [10], which comprise its posttranslational modification (PTM) pattern and its multiple interactions with cellular ligands such as other proteins, nucleic acids, lipids, co-factors, or salts, to name a few. Thus, target identification attempts to pinpoint potential interaction partners of a given compound, whereas target engagement measures if and to what extent the compound reaches and occupies its target in the cell [11]. The extent of intracellular target occupation can be determined by isothermal dose–response CETSA (ITDR-CETSA) experiments, which provide dose–response curves and compound potency in terms of EC50 values for the corresponding compound–protein interaction. While conventional CETSA experiments cover a specific temperature range (TR) to determine melting curves in the presence and absence of the compound, ITDR-CETSA experiments cover a specific compound concentration range (CCR) to determine the dose–response curves and potency of the compound at a given constant temperature. Importantly, unlike other methods for target engagement [11], CETSA can be performed on the undisturbed, natural system without potential bias due to labeling or modification of compounds or proteins, which has led to its broad application [6,10,11,12,13].

Next, the thermal proteome profiling (TPP, also termed MS-CETSA) method, a proteome-wide implementation of CETSA with multiplexed quantitative mass spectrometry [14,15] detection, was introduced [16,17,18]. Due to its unbiased detection of several thousands of proteins in parallel, the TPP method enables the identification of *hitherto* unknown (off-)targets of drugs or drug-like molecules in living cells, hence combining hypothesis-free target identification and target engagement in one analytical method [16,17,19,20,21,22,23,24,25,26,27,28].

One major challenge in TPP experiments in cells is to distinguish direct (primary) interactions from downstream effects of drug action (also referred to as indirect or secondary drug interactions), since both can result in thermal shifts of affected proteins [10]. While downstream effects contain additional functionally relevant information, they severely complicate the identification and engagement of primary targets. Aiming to distinguish between primary and secondary interactions, TPP studies using cell extracts have been suggested and performed in addition to TPP studies in living cells [16,18,19]. In cell extracts, the dilution of cellular contents with respect to living cells and the choice of lysis and buffer conditions are generally expected to result in impaired functional cellular integrity due to disruption of protein–protein interactions (PPI), loss of enzymatic activity, and a nonfunctional posttranslational modification machinery. For example, treatment of cells with dasatinib has been shown to affect the melting characteristics of the indirect BCR-ABL pathway targets CRK, CRKL, and SHIP2 (INPPL1), whereas no effect was observed in cell extract [16]. In another example [19], direct binding of JQ1 to SOAT1 has been confirmed by thermal stabilization in cell extracts, whereas other enzymes in the cholesterol synthesis pathway were not stabilized in cell extracts, suggesting that their stabilization in cell-based experiments is an indirect consequence of SOAT1 binding.

Furthermore, it has been shown that, independent of drug binding, the subunits of preferably large protein complexes such as the proteasome exhibit similar melting temperatures due to a phenomenon that has been termed thermal proximity coaggregation (TPCA) which was successfully applied to identify modulation of known protein complexes between different cell lines and types or in different phases of the cell cycle [10,29,30,31]. However, the predictive power for unknown PPI is rather limited [29] and a more definite allocation of proteins to unknown PPI, especially for protein complexes containing only a few subunits, is currently not possible due to the statistical nature of the method. Interestingly, small molecules can also influence TPCA: in a TPCA-like TPP study, the small molecule palbociclib (a CDK-4/6 inhibitor) has been shown to mediate stabilization of the entire 20S proteasome; however, this stabilization was not due to direct binding but was likely caused by ECM29 downregulation, for which the exact mechanism is not yet understood [32].

Based on these observations, we reasoned that a small molecule perturbation strategy could be applied to probe PPI under physiological conditions in living cells using isothermal dose–response characteristics. Similar to a TPCA experiment, where the complex partners exhibit similar melting behavior, we hypothesized that the thermal (de-)stabilization of a target protein by a small molecule and its concentration dependence is a common phenomenon among all protein complex partners and leads to similar dose–response characteristics. For testing our hypothesis, we employed a published model system [8] based on treatment of HL-60 cells with mitogen-activated protein kinase 14 (MAPK14; alternative name: mitogen-activated protein kinase p38 alpha, p38α) inhibitors AMG548 [33,34] and SB203580 [35] and considered the best-described and most closely linked MAPK14 interaction partners and phosphorylation targets MAP kinase-activated protein kinase 2 (MAPKAPK2) and MAP kinase-activated protein kinase 3 (MAPKAPK3) [36,37,38]. After testing our hypothesis using single protein detection by quantitative immunoblotting as described for the CETSA assay, we expanded the detection to the proteome wide scale using multiplexed quantitative mass spectrometry as described for TPP to identify *hitherto* unknown MAPK14 interaction partners or downstream targets by their similarity of dose–responses.

## 2. Results

Here, we employed a published CETSA model system based on treatment of HL-60 cells with MAPK14 kinase inhibitors [8]. In addition to the previously described MAPK14 immunodetection from living cells, we included the detection of MAPKAPK2/3 and performed CETSA experiments using HL-60 cell extract. Finally, we performed proteome-wide TPP experiments. An experimental overview is given in Supplementary Scheme S1.

### 2.1. CETSA—Thermal Stabilization of MAPK14 and Its Interaction Partners MAPKAPK2/3 upon Treatment with MAPK14 Inhibitors AMG-548 and SB203580

#### 2.1.1. CETSA Experiment in Living HL-60 Cells

First, we conducted CETSA experiments according to Jafari et al. [8]. In brief, HL-60 cells were treated with the MAPK14 inhibitors AMG-548 and SB203580, as well as the MAPK1/3 kinase inhibitor ERK 11e as negative control, aliquoted for each of the ten different temperature treatments, and the soluble (non-heat-denatured) protein fraction was obtained after mild cell lysis and centrifugation. Subsequent SDS-PAGE and immunoblotting were performed according to optimized conditions allowing for parallel detection of MAPK14, MAPKAPK2, MAPKAPK3, and GSK-3α (Supplementary Figure S1).

**Figure 1:**
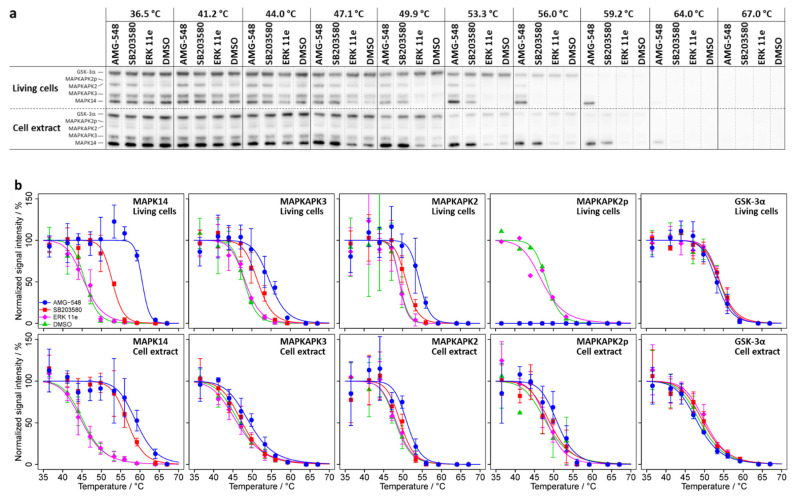
CETSA experiments for a temperature range between 36.5 and 67.0 °C without inhibitor (only DMSO) or at constant inhibitor concentrations of 20 µM, respectively. (**a**) Immunoblots for one of the replicates of CETSA experiments for living cells and cell extract, respectively. (**b**) Melting curves (solid lines) derived from integrated, normalized, and fitted signal intensities from the immunoblots. Datapoints and error bars represent means over the replicates +/− one standard deviation. Melting points and other parameters including the number of replicates are summarized in Table 1.

Our CETSA experiments for MAPK14 in living cells (Figure 1 and Table 1) resulted in different melting temperatures (*T_m_*) when treated with AMG-548 (*T_m_* = 60.6 ± 0.5 °C), SB203580 (*T_m_* = 52.9 ± 0.4 °C), or without inhibitor (*T_m_* = 45.8 ± 0.4 °C; DMSO control) and were in close agreement with the published [8] results (see Supplementary Table S1 for comparison). Thus, we obtained significant intracellular thermal stabilization of MAPK14 by AMG-548 (Δ*T_m_* = 14.8 °C, *p* = 4 × 10^−46^) and SB203580 (Δ*T_m_* = 7.0 °C; *p* = 4 × 10^−25^). As expected, the negative control inhibitor ERK 11e did not affect the thermal stability of MAPK14. Accordingly, for the negative control protein GSK-3α, neither inhibitor caused a thermal stabilization.

As suspected, the MAPK14 substrates and interaction partners MAPKAPK2 and MAPKAPK3 were also found to be thermally stabilized in living cells by AMG-548 (Δ*T_m_* = 5.2 °C, *p* = 5 × 10^−7^ and Δ*T_m_* = 6.6 °C, *p* = 4 × 10^−15^, respectively) and SB203580 (Δ*T_m_* = 1.8 °C, *p* = 0.18 and Δ*T_m_* = 3.5 °C, *p* = 2 × 10^−7^, respectively), but not by ERK 11e (Figure 1 and Table 1). The respective relative stabilizations (Δ*T_m_*) for MAPKAPK3 or MAPKAPK2 were less than half of those observed for MAPK14, with MAPKAPK2 appearing to be slightly less stabilized than MAPKAPK3. The SB203580-induced thermal stabilization of MAPKAPK2 was not statistically significant, however, treatment with SB203580, like with AMG-548, lead to a clear change in the abundance of non-phosphorylated MAPKAPK2 (Supplementary Figures S2 and S1b; see also the results for phosphorylated MAPKAPK2 below).

Finally, due to our optimized immunoblotting protocol, we were able to reveal a phosphorylation-dependent electrophoretic mobility shift (PDEMS) of MAPKAPK2, which appeared as a double band when living HL-60 cells were not treated with an inhibitor (DMSO control) or treated with ERK 11e (Supplementary Figure S1). In contrast, only a single band of MAPKAPK2 was detected after treatment with the MAPK14 kinase inhibitors AMG-548 and SB203580. The CETSA experiment showed no significant difference between the melting behavior of potentially phosphorylated MAPKAPK2 (MAPKAPK2p) and the dephosphorylated proteoform (MAPKAPK2) (DMSO control and ERK 11e treatment; Figure 1 and *p* > 0.05 in Supplementary Table S5), although the low immunoblot signal intensity for MAPKAPK2p only allowed for the acquisition of a single melting curve in each case. Since MAPKAPK2p was absent under AMG-548 or SB203580 treatment of living cells, no melting curves could be recorded for these conditions.

In summary, our CETSA experiment confirmed the observation of Jafari et al. [8] and showed highly significant temperature shifts for MAPK14 upon treatment of living HL-60 cells with the inhibitors AMG-548 or SB203580. In addition, we showed that the MAPK14 substrates and interaction partners MAPKAPK2 and MAPKAPK3 exhibit significant temperature shifts upon AMG-548 or SB203580 treatment, too, and that the MAPK14 kinase inhibitors AMG-548 and SB203580 inhibited apparent MAPKAPK2 phosphorylation.

#### 2.1.2. CETSA Experiment in HL-60 Cell Extract

For the differentiation of primary and secondary binding effects, CETSA experiments using cell extracts instead of living cells (cell extract test) are proposed in the literature. Therefore, we performed CETSA assays in HL-60 cell extracts under the same conditions as for living cells (Figure 1, Table 1, Supplementary Table S6). Again, we found a profound stabilization of MAPK14 in association with AMG-548 (Δ*T_m_* 13.6 °C, *p* = 6 × 10^−22^ in extract vs. 14.8 °C, *p* = 4 × 10^−46^ in cells) and SB203580 (Δ*T_m_* 11.3 °C, *p* = 3 × 10^−26^ in extract vs. 7.0 °C, *p* = 4 × 10^−25^ in cells) treatment. In the cell extract, as seen before in living cells, the negative control protein GSK-3α showed no significant change in melting behavior upon inhibitor treatment, and the negative control inhibitor ERK 11e also showed no effect on the proteins studied. As expected for secondary binding effects, the stabilization of MAPKAPK3 and MAPKAPK2 was abolished for SB203580 in the cell extract. However, the stabilization of MAPKAPK2 and MAPKAPK3 by AMG-548 was still observed (Δ*T_m_* 3.0 °C, *p* = 4 × 10^−4^ and 2.6 °C, *p* = 9 × 10^-7^).

**Figure 2:**
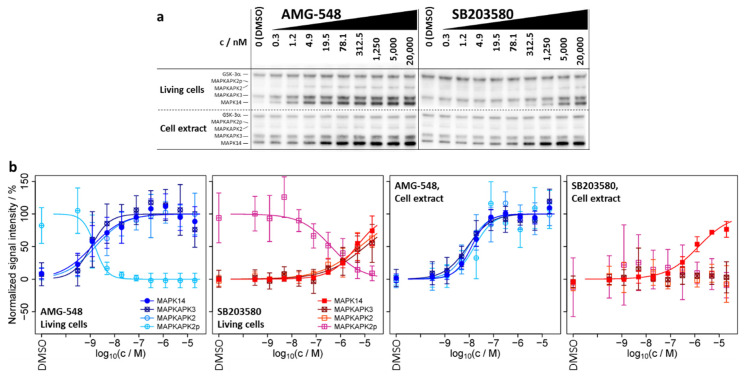
ITDR-CETSA experiments for a compound concentration range between 0.3 nM and 20 µM as well as without compound (only DMSO) at a constant treatment temperature of 51 °C. (**a**) Immunoblots for one of the replicates of ITDR-CETSA experiments for living cells and cell extract, respectively. (**b**) Dose–response curves (solid lines) derived from integrated, normalized, and fitted signal intensities from the immunoblots. Datapoints and error bars represent means over the four replicates (n = 4) +/− one standard deviation. The fitted pEC50 values are summarized in Table 2. Data for ERK 11e are shown in Supplementary Figure S3.

Notably, in the cell extract, MAPKAPK2 was detected as a double band irrespective of inhibitor treatment, which points to an inactivity of the dephosphorylation machinery under these conditions. As already observed in the living cell experiments, the potentially phosphorylated and dephosphorylated MAPKAPK2 proteoforms (MAPKAPK2p and MAPKAPK2) showed indistinguishable melting behavior (Figure 2 and respective *p*-values >> 0.05 in Supplementary Table S6). Consequently, parallel with MAPKAPK2, MAPKAPK2p was stabilized by AMG548 (Δ*T_m_* 2.8 °C, *p* = 0.014), but not by SB203580.

In summary, the CETSA experiments with living cells and cell extracts do not allow for clear conclusions regarding binding effects (direct or indirect inhibitor binding or the presence of PPI). There is no obvious similarity between the melting behavior of MAPK14 and its substrates MAPKAPK2/3, neither in absence nor in the presence of inhibitor, which does not suggest a relation between MAPK14 and MAPKAPK2/3. Based on the cell extract test, MAPK14 would be confirmed as a direct SB203580 binder (thermal stabilization in cells and extract) and MAPKAPK2/3 as indirect SB203580 binders (thermal stabilization only in cells), the latter suggesting co-stabilization by SB203580 due to PPI with MAPK14 in living cells. On the contrary, MAPK14 and MAPKAPK2/3 would be regarded as direct targets of AMG-548 (thermal stabilization in cells and extract), not suggesting any PPI between MAPK14 and MAPKAPK2/3.

### 2.2. ITDR-CETSA-MAPK14 and Its Substrates/Interaction Partners MAPKAPK2/3 Exhibit Nearly Identical Dose–Response Characteristics upon Kinase Inhibitor Treatment

Usually, ITDR-CETSA experiments are performed to obtain concentration-dependent characteristics of the small molecule–protein interactions. Here, we interrogated whether ITDR-CETSA can be used to more clearly differentiate between primary and secondary binding effects. Therefore, we performed ITDR-CETSA experiments at a constant treatment temperature of 51 °C, which was selected from the melting curves in order to obtain the largest possible average difference in signal intensity for the proteins between treatment with the inhibitor and the DMSO control, and at a range of compound concentrations for the three inhibitors shown in Figure 2. The ITDR-CETSA experiments were again performed in living cells as well as in cell extracts (Figure 2 and Table 2). We found that, in the cell extract, AMG-548 exhibited a lower potency for MAPK14 stabilization than in living cells, as expressed by the lower pEC50 (negative decadic logarithm of the EC50 value in molar concentration units) of 7.9 in the extract, compared to 8.9 in living cells. On the contrary, SB203580 showed increased stabilization potency in the extract (pEC50 = 5.9) when compared to living cells (pEC50 = 5.3), suggesting different cellular uptake/export or metabolism parameters for the two inhibitors.

From our perspective, the most interesting finding was that MAPK14 and its substrates MAPKAPK2/3 exhibit a high similarity in pEC50 values and dose–response characteristics upon treatment of living cells with AMG-548 (pEC50 = 8.9 ± 0.2, 9.0 ± 0.2, 8.8 ± 0.2, 8.8 ± 0.1 for MAPK14, MAPKAPK3, MAPKAPK2, and MAPKAPK2p, respectively) and SB203580 (pEC50 = 5.3 ± 0.1, 5.0 ± 0.3, 5.2 ± 0.2, 6.4 ± 0.2, respectively), as well as for AMG-548 in cell extracts (pEC50 = 7.9 ± 0.1, 8.1 ± 0.1, 8.0 ± 0.2, 7.8 ± 0.2, respectively) (Figure 2b and Table 2). For SB203580 in the cell extract, binding characteristics were only accessible for MAPK14 due to the lack of significant changes in melting behavior for MAPKAPK2/3, which was already observed in the melting curves (see Section 2.1.2). For the same reason, the negative control inhibitor ERK 11e did not cause dose–responses for the assayed proteins and conditions (Supplementary Figure S3).

Based on our results for MAPK14 and its substrates/interaction partners MAPKAPK2 and MAPKAPK3, exhibiting different melting but similar dose–response behavior, we propose that ITDR-CETSA experiments allow differentiation between primary and secondary binding effects and provide a better and more reliable source of information about the presence of intracellular PPI than the “cell extract test” or TPCA, and we called this method small molecule arranged thermal proximity coaggregation (smarTCPA).

### 2.3. Determination of Candidate Interaction Partners of MAPK14 in Living HL-60 Cells by Means of smarTCPA

**Figure 3:**
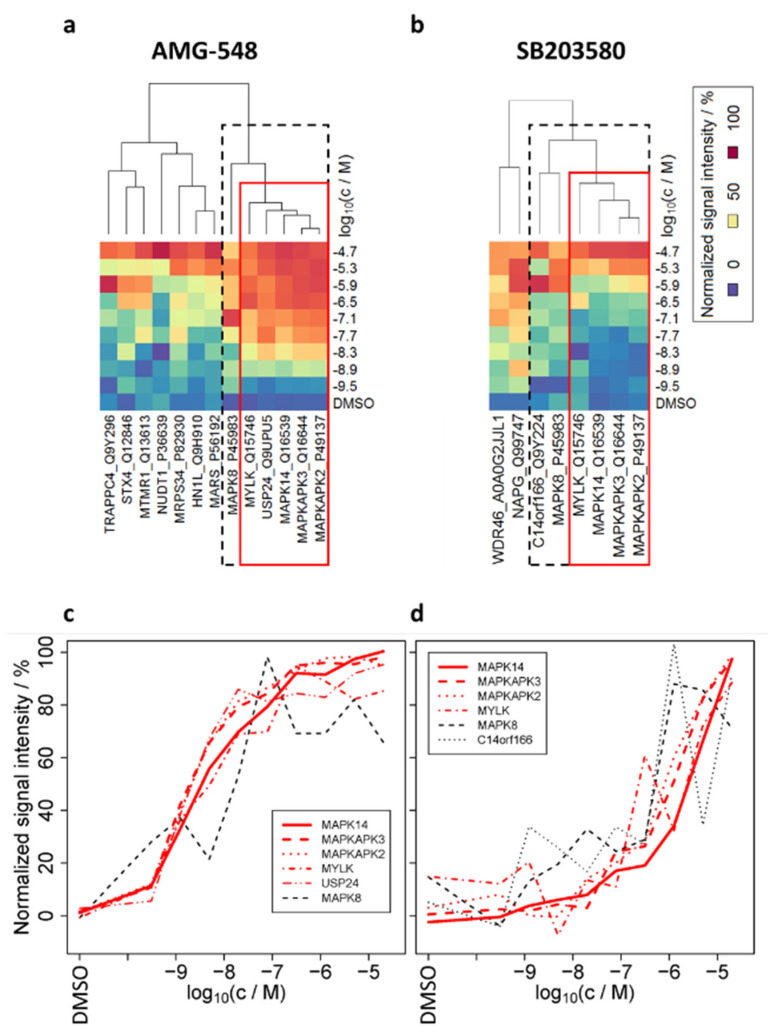
Heat maps after cluster analysis of the filtered (acceptable fit of dose–response curves for stabilized proteins, see experimental section for filter criteria) dose–responses (mean over replicates), derived from TPP-CCR experiments in living cells. (**a**) AMG-548, (**b**) SB203580. Narrower and wider clusters around the primary target MAPK14 are represented by red and dashed boxes, respectively. See Supplementary Figure S4 for the same representation for ERK 11e. Plots (**c**,**d**) show the normalized signal intensities of the proteins highlighted by the red and dashed boxes in (**a**,**b**), respectively, which are plotted in two dimensions instead of color-coded.

As we showed that similar dose–responses are informative to differentiate between primary and secondary binding effects (here: PPI), we were curious to reveal if our findings could be transferred to a proteome-wide approach and give access to novel PPI candidates in living cells. Therefore, we performed TPP with compound concentration range (TPP-CCR) experiments according to published procedures [18,23]. In brief, after preparing tryptic peptides from the non-denatured protein fractions (used for immunoblotting in the previous sections) applying the SP3 method [39], the peptides were labeled using TMT10plex, coding for inhibitor concentration, and the samples belonging to a TMT set were combined, offline high-pH fractionated, and analyzed by MS. After filtering the identified 4563 protein groups for acceptable stabilizing dose–response characteristics, 13, 8, and 24 proteins were obtained for AMG-548, SB203580, and ERK 11e treatment, respectively (Figure 3, Supplementary Figure S4, Table 3, and Supplementary Table S3).

Phosphorylated proteins (such as phosphorylated MAPKAPK2) could not be distinguished from their non-phosphorylated proteoforms in the present MS analysis. For ERK 11e, good fits (pseudo *R*^2^ > 0.8) were obtained for its target protein MAPK3 [8,40] (pEC50 = 6.4) (alternative gene name ERK1, see Supplementary Figure S5b for the dose–response curve) and four other proteins.

**Figure 4:**
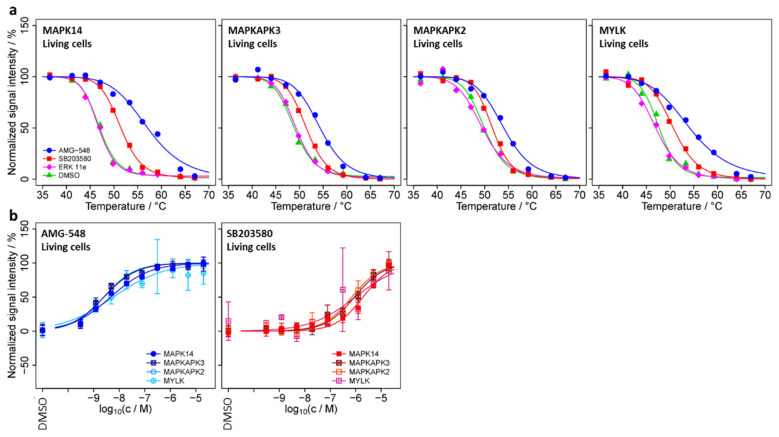
Melting curves (**a**) and dose–response curves (**b**) for selected proteins from the TPP-TR and TPP-CCR experiments.

The EC50 values for MAPK14 (8.33 ± 0.03, 5.71 ± 0.08) and its substrates MAPKAPK2 (8.60 ± 0.08, 6.15 ± 0.06) and MAPKAPK3 (8.60 ± 0.05, 6.05 ± 0.08) upon treatment with AMG-548 and SB203580, respectively, agreed with the results of our ITDR-CETSA experiments (see above). By cluster analysis (Figure 3a,b), we confirmed the high similarity of the MAPK14, MAPKAPK2, and MAPKAPK3 dose–responses (Figure 3c,d and fitted dose–response curves in Figure 4b) upon treatment with AMG-548 as well as SB203580 in the present smarTCPA analysis. Additionally, MAPK14, MAPKAPK2, and MAPKAPK3 exhibited the best reproducibility and fits to the dose–response model of the current TPP-CCR study (*pseudo R*^2^ > 0.9, respectively). Moreover, it is striking that additional proteins exhibited similar dose–responses upon treatment with AMG-548 (MYLK, USP24, and MAPK8) and SB203580 (MYLK, MAPK8, and C14orf166) and, therefore, are intracellular candidate interaction partners of MAPK14. Furthermore, a ranking based on the similarity of their dose–responses to those of MAPK14 and their confirmation by both MAPK14 inhibitors (AMG-548 and SB203580) leads to MYLK as being the candidate MAPK14 interaction partner with the highest confidence, followed by MAPK8 (alternative gene name JNK1). We regard USP24 and C14orf166 as potential MAPK14 interactors with lower confidence, because their dose–response similarity to MAPK14, which was obtained with one inhibitor, could not be confirmed by the respective other inhibitor.

In summary, we have shown that proteome-wide smarTCPA/TPP-CCR experiments not only give access to pEC50 values of individual small-molecule-binding proteins, but also allow for the interrogation of secondary binding effects such as PPI. Due to their similar dose–response characteristics, we not only confirmed well-known interaction partners (MAPKAPK2 and MAPKAPK3) of MAPK14, but also found candidate proteins which have not been described as interaction partners of MAPK14, yet. Based on our findings, we support the idea that direct binders generally can be distinguished by their different inhibitor specificity profile (different binding strengths to different inhibitors), while co-stabilized proteins should closely resemble the inhibitor specificity profile of the direct binder.

### 2.4. TPP-TR Confirmation Experiment

In principle, the changes in signal intensity observed in TPP-CCR experiments may be due to both thermal stabilization and/or a change in abundance of the candidate protein ([41]). We performed a single set (no replicates) of temperature-range TPP experiments (TPP-TR) to test for thermal stabilization of the proteins with acceptable stabilizing dose–response characteristics in the TPP-CCR experiments, as shown in Figure 3. All of these proteins were identified in the TPP-TR experiments except for STX4, WDR46, and the potential MAPK14 interactor MAPK8, whose thermal stabilization could, therefore, not be assessed. The obtained melting curves (Supplementary Figure S6) showed clear AMG-548- or SB203580-induced thermal stabilizations for MAPK14, MAPKAPK3, MAPKAPK2, and MYLK (Figure 4a), indicating that the intensity changes observed for these proteins in the TPP-CCR experiments were due to thermal stabilization and not to a change in abundance. No clear thermal stabilization was observed for the other proteins, including the lower confidence potential MAPK14 interactors USP24 and C14orf166. In addition, thermal stabilization induced by ERK 11e was observed for its known target MAPK3 (Supplementary Figure S5a).

Taken together, the results of the TPP-CCR and TPP-TR experiments suggest that MYLK—along with the known MAPK14 interactors MAPKAPK2/3—is an intracellular interaction candidate of MAPK14 and that smarTPCA may be useful for detecting novel PPI in living cells, for which we—in analogy to target engagement—propose the term PPI engagement.

## 3. Discussion

In recent years, CETSA and its proteome-wide extension, TPP, have proven to be effective tools for identifying interactions of small molecules with their target proteins as well as with off-targets in living cells. Here, we asked the question whether dose–response characteristics obtained by ITDR-CETSA or TPP-CCR experiments can be exploited to reveal secondary effects downstream of the primary binding event, such as changes in PTM or PPI. Therefore, we investigated a well-studied model system of HL-60 cells treated with the MAPK14 inhibitors AMG-548 and SB203580, for which thermal stabilization of the primary target (MAPK14) has been previously reported [8,42].

In our CETSA experiments, we confirmed thermal MAPK14 stabilization upon AMG-548 and SB203580 treatment of living cells (Table 1 and Supplementary Table S1). The thermal stabilization of MAPK14 by AMG-548 was slightly reduced in the cell extract compared with living cells (−1.16 °C, Table 1), whereas treatment with SB203580 caused a stronger thermal shift of MAPK14 in the cell extract than in living cells (+4.28 °C, Table 1). The latter might be due to a lower local SB203580 concentration within cells, e.g., because of a low cell permeability of SB203580, which is also reflected in the apparently tighter binding (expressed by the higher pEC50 values, Table 2) of SB203580 to MAPK14 in the cell extract when compared with living cells. Although the molecular details are largely unknown, other reasons for the altered thermal shifts may be due to the different protein contexts and concentrations in extract and cells.

We also noted that an absolute comparison of pEC50 values from different studies is inappropriate (Table 2, Supplementary Tables S2 and S7) because ITDR-CETSA results are known to be highly dependent on experimental conditions, and thus are often referred to as isothermal dose–response fingerprints [7,8,42].

### 3.1. Phosphorylation Has No Effect on the Thermal Stabilisation of Phospho-MAPKAPK2

Due to our chosen experimental CETSA set-up, we were able to follow the role of phosphorylation on the thermal stability of MAPKAPK2 (Figure 1 and Supplementary Figures S1 and S2). The phosphorylation of MAPKAPK2, a long-known, direct downstream phosphorylation target of MAPK14 [36], was revealed by a PDEMS [43] towards apparent higher molecular weight. This PDEMS has been well described for MAPKAPK2 in numerous publications (Figure 5 in [44]; Figure 3 in [45]; Figure 2A in [46]; Figure 7E in [47]; Figure 10B in [48]). Further confirmation of MAPKAPK2 phosphorylation is based on our observations in the context of kinase inhibitor treatments (AMG-548, SB203580, or ERK 11e) of living HL-60 cells or cell extracts. In living cells, treatment with both MAPK14 kinase inhibitors (AMG-548, SB203580) showed a rapid and active dephosphorylation machinery, as MAPKAPK2 was observed only as a single band after immunodetection (dephosphorylated MAPKAPK2). This was also confirmed by an increase in the signal intensity of dephosphorylated MAPKAPK2 after treatment with the MAPK14 kinase inhibitors (AMG-548, SB203580). Thus, phosphorylation of MAPKAPK2 to the basal level seems to be obstructed by inhibition (by AMG-548 or SB203580) of MAPK14 in living HL-60 cells, which has been described earlier for SB203580 treatment of MDCK cells (Figure 3 in [49]) or of Dengue virus-infected mice (Figure 12 in [50]). In cell extracts, phospho-MAPKAPK2 (MAPKAPK2p) signals were observed irrespective of inhibitor treatment, suggesting an inactive dephosphorylation machinery due to cell lysis conditions and homogenization.

Interestingly, in the context of our CETSA experiments, the phosphorylated and dephosphorylated MAPKAPK2 (MAPKAPK2p and MAPKAPK2) exhibited the same melting characteristics under the same treatment conditions, respectively (Figure 1 and respective *p* values >> 0.05 in Supplementary Table S6). This finding is consistent with the reported lack of effect on melting behavior for the vast majority of phosphosites: Although the impact of protein phosphorylation on protein thermal stability is still under investigation and subject of a current scientific debate, it seems that influences of phosphorylation on thermal protein stability are rather low, and that only 1.5–3% of the phosphosites lead to a significant thermal shift [51,52,53,54].

In the context of our ITDR-CETSA experiments (Figure 2b), two effects contributed to the observed increase in MAPKAPK2 dose–response signal intensity upon AMG-548 and SB203580 treatment of living cells (at 51 °C treatment temperature): first, the thermal stabilization described above and observed in the respective melting curves (Figure 1b; a melting curve shift to higher temperatures leads to an increase in signal intensity at or near the melting point) and, second, an increase in MAPKAPK2 abundance due to dephosphorylation of the basal levels of MAPKAPK2p by intracellular phosphatases (Supplementary Figure S2). In conclusion, intracellular AMG548 and SB203580 binding and inhibition of MAPK14 appear to be directly correlated with both MAPKAPK2 co-stabilization and MAPKAPK2 abundance increase, providing the opportunity to link not only co-stabilization but also downstream processes to the primary binding event through the similarity of dose–response characteristics by smarTPCA.

### 3.2. CETSA in Cell Extract Is Not Universally Applicable for the Interrogation of Primary and Secondary Binding Effects

In our CETSA experiment with living cells, we observed thermal stabilization of MAPK14 as well as MAPKAPK2 and MAPKAPK3 after treatment with AMG-548 and SB203580. Since, to our knowledge, no direct binding of the MAPK14 inhibitors AMG-548 and SB203580 to MAPKAPK2 or MAPKAPK3 has been reported (SB203580 has even been shown to be inactive towards MAPKAPK2/3, see Section 3.4. below), we concluded that secondary interactions are most likely the cause of the observed thermal MAPKAPK2/3 stabilization. Therefore, we performed CETSA in cell extracts, which has been proposed to allow distinguishing between primary and secondary interactions [16,18,19]. Interestingly, only for SB203580, we confirmed MAPK14 as direct and MAPKAPK2/3 as indirect binders by their presence and absence of thermal stabilization in cell extract, respectively. In contrast, for AMG-548, in addition to the expected thermal shifts for MAPK14, we still detected shifts for MAPKAPK2 and MAPKAPK3 in cell extracts. Thus, MAPKAPK2 and MAPKAPK3 would be misclassified as direct AMG-548 binders.

This suggests that intracellular PPI responsible for co-stabilization of secondary small-molecule binders may still be present in the extract and may lead to misclassification of secondary interactors as primary interactors. We therefore conclude that CETSA with cell extract is not suitable to discriminate between primary and secondary interactors in all cases and that further experiments are needed to avoid misclassification.

### 3.3. smarTPCA—A Novel Approach to Interrogate Primarry and Secondary Binding Effects by Means of Dose–Reponse Charateristics

Next, we investigated whether dose–response characteristics are better suited for distinguishing primary and secondary binding effects. By our own experiments, we found that MAPKAPK3 and MAPKAPK2, the known interaction partners of MAPK14, both have similar dose–response characteristics to MAPK14 itself. This finding was in part supported by the original TPP study by Savitski and colleagues, who reported similar dose–response characteristics for the BCR-ABL1 interaction partners CRK and CRKL upon the treatment of living K562 cells with dasatinib (Supplementary Figure S11a in [16]), however, no dose–response curve was obtained for the primary dasatinib target BCR-ABL1 itself.

Finally, we were able to extract similar dose–response characteristics for interacting proteins from earlier published experiments, supporting our observation. In a TPP study using living K562 cells, the small molecule Na⁺/K⁺-ATPase (Na⁺/K⁺ pump) inhibitor ouabain induced very similar dose–responses of the α-subunit (ATP1A1) and the β-subunit (ATP1B3) of the Na⁺/K⁺ pump (Supplementary Figure S6c in [20]). The dose–response similarity was not mentioned by the authors, and while crystal structures and other studies show that ouabain directly binds to ATP1A1 [55,56,57], ATP1B3 must be considered as an indirect co-stabilized target protein of ouabain.

Another TPP study in living K562 cells showed, although not mentioned by the authors, similar dose–responses of histone deacetylases 1 and 2 (HDAC1, HDAC2) and mesoderm induction early response protein 1 (MIER1) upon treatment with the small molecule non-selective histone deacetylase inhibitor panobinostat [18]. As a known HDAC1/2 complex member, MIER1 was regarded as secondary panobinostat target [58]. Additionally, we inferred from the provided supplementary 2D-TPP datasets of a panobinostat follow-up study [21] that MIER1 was stabilized by Panobinostat—again with similar dose–responses when compared to HDAC1 and HDAC2—in living HepG2 cells (Suppl. Dataset1 in [21]) but not in HepG2 cell extract (Suppl Dataset2 in [21]), suggesting that the MIER1/HDAC1/2 complex was not stable under the chosen lysis and extraction conditions. Also, in a tissue TPP study [59], MIER1 was stabilized by panobinostat and its dose–responses that we extracted from the respective supplementary dataset (Supplementary Data 12 in [59]), were again similar to those of HDAC1 and HDAC2.

In summary, based on our results and reanalysis of the available data, we are confident that the analysis of dose–response characteristics will—in addition to CETSA or TPP in cell extract—provide another level of evidence for characterizing primary and secondary binding effects. For this purpose, we propose small molecule treatment in combination with dose–response similarity analysis from ITDR-CETSA or TPP-CCR experiments as a novel method, which we have termed small molecule arranged thermal proximity coaggregation (smarTPCA).

### 3.4. Application of Small Molecule Arranged Thermal Proximity Coaggregation (smarTPCA) in a Proteome-Wide TPP-CCR Experiment

As we showed that similar dose–response characteristics are the key to distinguish primary and secondary binding effects, we were interested to apply smarTPCA in a proteome-wide TPP-CCR approach. In addition to the already known MAPK14 interaction partners MAPKAPK2 and MAPKAPK3, we revealed that MAPK8 and MYLK had similar dose–responses to MAPK14 upon treatment with AMG-548 and SB203580.

A comprehensive study on kinase inhibitor selectivity has shown high activity of SB203580 against MAPK14, but inactivity against MAPK8 and MYLK, as well as MAPKAPK2 and MAPKAPK3 (Supplementary Table S3 in [60]). High activity of SB203580 towards MAPK14 (K_D_ = 12 nM) and its inactivity towards MAPKAPK2 and MYLK has also been found in another study (Supplementary Table S4 in [61]), in which SB203580 has been reported to bind MAPK8 with mediocre affinity (K_D_ = 1.1 µM; MAPKAPK3 has not been tested).

For AMG-548, most of the kinase inhibition data are undisclosed. About 35 undisclosed kinases and seven kinases with their inhibitory constants (K_i_) for AMG-548 have been reported, whereof MAPK8 exhibits a low affinity (K_i_ = 11,480 nM) in contrast to MAPK14 with high affinity (K_i_ = 0.5 nM) [34]. Therefore, we assume that the EC50 of MAPKAPK2, MAPKAPK3, MAPK8, and MYLK in the low nanomolar range as found in our TPP-CCR experiment upon treatment with AMG-548, is based on co-stabilization by PPI with MAPK14 instead of direct binding, and that the same mechanism applies for SB203580.

Thus, with the confirmation of known and the discovery of novel intracellular protein interaction partners of MAPK14, we are convinced that smarTPCA is a meaningful extension of thermal proximity coaggregation (TPCA) by integrating small molecule perturbation strategies to reveal PPI in living cells. The observed similar dose–responses of complex members go in line with the observation in yeast that entire complexes appear to be essential for cell survival (modular essentiality) and that members of the same protein complex respond in a coherent fashion to the same drugs ([62,63]).

Interestingly, of the 22 MAPK14-interacting human proteins listed in the uniprot database (release 2021_03, Supplementary Table S4), ten were identified by MS in the present smarTPCA/TPP-CCR study (underlined in Supplementary Table S4) but only MAPKAPK2/3 showed acceptable dose–response characteristics upon treatment with the two MAPK14 inhibitors (underlined and bold in Supplementary Table S4; MAPK3 showed acceptable dose–response characteristics for ERK 11e but not for AMG-548 or SB203580). At this stage, we cannot rule out the possibility that the relatively low number of confirmed interaction partners is an indication of the low sensitivity of smarTPCA, or is due to the fact that only a small number of MAPK14 PPI are actually present (in HL-60 cells) and stable under the conditions of the analysis. Further studies are needed to benchmark the robustness of smarTPCA and how stability of interactions, as well as stoichiometry, affect the dose–responses of complex members.

In summary, our smarTPCA approach offers the opportunity to identify PPI in living cells, which could be referred to as PPI engagement by analogy with target engagement of small molecules.

## 4. Materials and Methods

### 4.1. Sample Preparation

#### 4.1.1. Sample Preparation from Living Cells

The preparation of the non-heat-denatured protein samples was based on the protocols by Jafari et al. [8] and Franken et al. [18] (see Supplementary Table S7 for comparison).

##### CETSA and TPP-TR

*Compound treatment:*

HL-60 cells (ATCC No. CCL-240) were expanded to about 10^7^ cells in 80 mL cell culture medium (RPMI-1640 supplemented with 10% (*v*/*v*) FBS, 1% (*v*/*v*) penicillin-streptomycin and 1% (*v*/*v*) l-glutamine solution) and divided into four aliquots (20 mL each, 25 million cells per flask). 40 μL of 10 mM stock solutions of the three different kinase inhibitors AMG-548 (Tocris, cat. no. 3920), SB203580 (Tocris, cat. no. 1202), or ERK 11e (VX-11e; Tocris, cat. no. 4465) in dimethyl sulfoxide (DMSO) were added separately to three of the aliquots to obtain a final concentration of 20 μM per compound. The same volume of DMSO was added to the remaining aliquot serving as the vehicle (DMSO) control. After incubation (1 h, 37 °C, 5% CO_2_, in cell culture CO_2_ incubator), the cell suspensions were transferred into 50 mL conical tubes, cell number and viability were determined, and after centrifugation (300 relative centrifugal force (rcf), 3 min, room temperature (r.t.)), the supernatants were completely removed and discarded. The cells were washed using 20 mL Dulbecco’s phosphate-buffered saline (PBS), respectively, by resuspension and centrifugation (300 rcf, 3 min, r.t.). After the complete removal of the supernatants, the cells were transferred into pre-weighed 2 mL Eppendorf tubes using 0.5 mL PBS (r.t.), respectively, for resuspending. After centrifugation (300 rcf, 3 min, r.t.) and removing as much as possible of the supernatants, the cell wet weights were determined.

*Temperature treatment:*

Each cell pellet was resuspended in 1 mL ice-cold PBS supplemented with a protease inhibitor cocktail (cOmplete ULTRA Tablets, Mini, EDTA-free, EASYpack, Roche, Basel, Switzerland) and aliquoted into 10× 100 µL (∼2.5 million cells) into PCR tube strips in a way that the four different samples were placed together in one strip, respectively, to ensure side-by-side comparability (10× strips of 4 PCR tubes: AMG-548, SB203580, ERK 11e, and DMSO control). The suspensions were shortly centrifuged (1 s or less) using a benchtop centrifuge that was equipped with an inset for PCR tubes to release trapped air and to achieve an even liquid level without pelleting the cells. The 100 µL cell suspensions were then temperature treated by a 7 min pre-incubation at r.t., a 3 min incubation at the following different temperatures in the PCR cycler (DNA Engine Tetrad 2, lid temperature 70 °C, gradient block 1: 36–55 °C, gradient block 2: 48–67 °C), and a 3 min post-incubation at r.t. in a metal heating block for uniform heat dissipation.
PCR strip:12345678910Treatment temp./°C:36.541.244.047.149.853.356.059.264.067.0

Afterwards, the samples were snap frozen in liquid nitrogen and stored at −80 °C or processed directly.

*Preparation of cell extracts containing the fraction of soluble, non-denatured proteins:*

The cells were lysed by four freeze/thaw cycles consisting of thawing the samples (in a heat block set to 25 °C and shaking at 1250 rpm) accompanied by mixing them several times by inversion and then shock freezing them again in liquid nitrogen. The resulting lysates were kept cold (on ice or 4 °C) from then on. After centrifugation (20,000 rcf, 30 min, 4 °C) to pellet the cell debris together with precipitated and aggregated proteins, the supernatants were recovered and, after determining the total protein concentration (Pierce 660 nm Protein Assay, BSA as standard), the resulting cell extracts containing the fraction of soluble, non-denatured proteins were shock frozen in liquid nitrogen and stored at −80 °C.

##### ITDR-CETSA and TPP-CCR

HL-60 cells were expanded as described above for CETSA and TPP-TR to 1.25 million cells per milliliter. 4 μL, respectively, of a 4-fold dilution series of the three kinase inhibitors (AMG-548, SB203580, and ERK 11e: 10, 2.5, 0.625, 0.156, 0.0391, 0.00977, 0.00244, 0.000610, 0.000153 mM, and vehicle (DMSO)) were transferred to 2 mL Eppendorf tubes labeled with the corresponding designated final concentrations (20, 5, 1.25, 0.313, 0.0781, 0.0195, 0.00488, 0.00122, 0.000305 μM, and vehicle (DMSO)). A number of 2.5 million cells in 2 mL of medium were added to each tube and incubated (1 h, 37 °C). After centrifugation (300 rcf, 3 min, r.t.) and removing all of the supernating culture medium, the cells were washed once with 2 mL and once with 0.5 mL PBS (r.t.) by gently resuspending the cells, followed by centrifugation (300 rcf, 3 min, r.t.) and the complete removal of the supernatants.

After transferring the cells from the 2 mL Eppendorf tubes into correspondingly labeled PCR tubes (tube strips) using 100 µL of ice-cold PBS supplemented with protease inhibitors for resuspending the cells, the samples were further processed as described above for CETSA and TPP-TR, however, by applying a single treatment temperature of 51 °C.

#### 4.1.2. Sample Preparation from Cell Extract

*Preparation of Cell Extracts from Untreated Cells:*

HL-60 cells were expanded as described above for CETSA and TPP-TR to about 1.25 million cells per ml. 50 mL of the cell suspension, respectively, was transferred into 50 mL conical tubes. After centrifugation (300 rcf, 3 min, r.t.) and removing all of the supernating culture medium, the cells were washed twice with 25 mL PBS (r.t.) by gently resuspending the cells, followed by centrifugation (300 rcf, 3 min, r.t.) and the removal of the supernatant. The cells were transferred into pre-weighed 2 mL LoBind Eppendorf tubes using 1 mL PBS (r.t.), respectively, for resuspending. After centrifugation (300 rcf, 3 min, r.t.) and removing as much as possible of the supernatants, the cell wet weights were determined. The cells were stored at −80 °C after shock-freezing in liquid nitrogen.

Cell extracts were obtained by four freeze/thaw cycles as described above for CETSA and TPP-TR using 10 µL ice cold PBS supplemented with protease inhibitors per milligram of cell pellet wet weight for the initial resuspension of the pellet, followed by centrifugation (20,000 rcf, 30 min, 4 °C), and the recovery of the supernatant. After determining the protein content, the cell extracts were diluted to 1.8 mg/mL total protein concentration using ice-cold PBS supplemented with protease inhibitors and aliquots of 99 µL in PCR tube strips, and 1.1 mL in LoBind 1.5 mL Eppendorf tubes were shock-frozen in liquid nitrogen and stored at −80 °C or directly processed.

##### CETSA

*Compound and temperature treatment as well as preparation of cell extracts containing the fraction of soluble, non-denatured proteins:*

2.2 μL, respectively, of the same 10 mM stock solutions of the three kinase inhibitors AMG-548, SB203580, and ERK 11e in DMSO, used for treatment of living cells, were added to three of the 1.1 mL aliquots of 1.8 mg/mL HL-60 cell extract. A fourth aliquot of 1.8 mg/mL HL-60 cell extract was supplemented with 2.2 µL of DMSO. After mixing and incubation (1 h, 37 °C), the four extracts were distributed into 100 µL each in PCR tube strips, so that the samples to be compared were placed together in one strip (10× strips of 4 PCR tubes: AMG-548, SB203580, ERK 11e, and DMSO control). After a brief centrifugation using a benchtop centrifuge, the temperature treatment at the ten different temperatures was performed as described in Section 4.1.1.

Additionally, the preparation of cell extracts containing the fraction of soluble, non-denatured proteins was performed as described in Section 4.1.1. for living cells, but omitting the freeze/thaw steps for cell lysis.

##### ITDR-CETSA

*Compound and temperature treatment as well as preparation of cell extracts containing the fraction of soluble, non-denatured proteins:*

In order to maintain the same final DMSO concentration of 0.2% (*v*/*v*) in the samples, the same 4-fold dilution series of the three kinase inhibitors (AMG-548, SB203580, and ERK 11e: 10, 2.5, 0.625, 0.156, 0.0391, 0.00977, 0.00244, 0.000610, 0.000153 mM, and vehicle (DMSO)) that were used for the treatment of living cells were pre-diluted 5-fold with ddH2O, resulting in concentrations of 2, 0.5, 0.125, 0.0313, 0.00781, 0.00195, 0.000488, 0.000122, 0.0000305 mM, and vehicle (20% *v*/*v* DMSO). Of each of these kinase inhibitor solutions, 1 µL was added to 30 (corresponding to the three kinase inhibitors with nine different concentrations plus vehicle control, respectively) of the 99 µL aliquots of 1.8 mg/mL HL-60 cell extract, resulting in the same final inhibitor concentrations as for the treatment of living cells (20, 5, 1.25, 0.313, 0.0781, 0.0195, 0.00488, 0.00122, 0.000305 μM, and vehicle (DMSO)). After mixing, incubation (1 h, 37 °C), and a brief centrifugation using a benchtop centrifuge, the temperature treatment at a single temperature of 51 °C was performed as described in Section 4.1.1.

Additionally, the preparation of cell extracts containing the fraction of soluble, non-denatured proteins was performed as described in Section 4.1.1. for living cells, but omitting the freeze/thaw steps for cell lysis.

### 4.2. Protein Detection and Quantification

#### 4.2.1. Detection of Selected Proteins by Immunoblotting after SDS-PAGE Separation

*SDS-PAGE:*

For each sample set comprising either ten temperature range samples or ten compound concentration range samples, the same volume of the cell extracts containing the fraction of soluble, non-denatured proteins was used for further analysis. This volume was determined using the two lowest temperature samples (36.5 and 41.2 °C) per temperature range sample set so that they contained an average of 5 µg total protein (e.g., 2.78 µL for all temperature range samples of a sample set whose two lowest temperature samples contain an average of 1.8 mg/mL protein). For the compound concentration range experiments, all samples of a set were used for averaging. The calculated volume was respectively transferred into PCR tubes of a tube strip, supplemented with SDS sample buffer (final concentrations: 25 mM dithiothreitol (DTT), 7.5% glycerol, 3% SDS, 37.5 mM Tris/HCl pH 7.0, 0.005% bromophenol blue), incubated for 10 min at r.t. and applied to SDS-PAGE under external cooling in an ice bath (MW marker: PageRuler pre-stained protein ladder (Thermo scientific, Waltham, MA, USA); NuPAGE 4–12% Bis-Tris Gels, 1.0 mm, Midi Protein Gel, 26-well (Thermo fisher scientific); ice cold MOPS SDS running buffer (50 mM MOPS, 50 mM Tris base, 0.1% SDS, 0.003% EDTA (free acid)); 50 V for 15 min, then 200 V for about 1.5 h, i.e., the rest of the run).

*Immunoblotting:*

Immunoblotting was performed at 2.5 A and 25 V for 7 min on isopropanol-activated PVDF membranes that were pre-incubated in anode buffer (300 mM Tris, 100 mM Tricine, pH 8.7) using the TransBlot Turbo Transfer System (BioRad, Hercules, CA, USA) with re-used filter papers (special tissue, cleaned extensively with ddH2O after each run) from transfer packs soaked in anode buffer or cathode buffer (300 mM aminocaprionic acid, 30 mM Tris, pH 8.6), respectively. After blotting, the membrane was washed with ddH2O, Ponceau S-stained (0.1 % Ponceau S in 5 % acetic acid, background destaining with 5 % acetic acid) for blot quality control, destained with TBS, and blocked for 0.5 h using blocking solution (2 % BSA and 3 % skim milk powder in TBS with 0.1% *w*/*v* Tween20 (TBST)) under shaking. Simultaneous incubation with primary antibodies (α-MAPK14 (1:2500; anti-p38alpha/beta, mouse, monoclonal, Santa Cruz, cat. no. sc-7972), α-MAPKAPK3 (1:1300; anti-3pK, mouse, monoclonal, Santa Cruz, cat. no. sc-365148), α-MAPKAPK2 (1:50; mouse, monoclonal, Santa Cruz, cat. no. sc-393609), and α-GSK-3α (1:1000; rabbit, monoclonal, Cell Signaling, cat. no. D80E6); optimized dilutions) was performed in blocking solution overnight at 4 °C in a sealed bag without shaking. The membrane was washed three times for 10 min with TBST and simultaneous incubation with secondary antibodies (anti-mouse (1:5000; Cell signaling, cat. no. 7076S) and anti-rabbit (1:5000; Cell signaling, cat. no. 7074S), HRP conjugates, from goat) was performed in blocking solution for 1 h at r.t. under shaking. After washing the membrane (4× 10 min TBST, 1× 10 min TBS), ECL signals were detected using the Clarity Max Western ECL substrate (Bio-Rad, cat. no. 1705062) in the ChemiDoc MP Imaging System (BioRad, Hercules, CA, USA) at high resolution (1 × 1). ECL signal intensities were integrated from “raw16.tif” image files separately for each lane using the “Analyze → Gels” function from the ImageJ software version 1.52a.

#### 4.2.2. Protein Analysis by LC-MS/MS

##### TPP-TR

*Short SDS-PAGE:*

For each sample set comprising ten temperature range samples, the same volume of the cell extracts containing the fraction of soluble, non-denatured proteins was used for further analysis. Volume calculation for 30 µg total protein was performed as described for SDS-PAGE and immunoblotting. The calculated volume was respectively transferred into 0.5 mL LoBind Eppendorf tubes and evaporated to dryness using vacuum centrifugation (SpeedVac concentrator, Eppendorf, Hamburg, Germany). The dried proteins were each reconstituted in 10 µL of SDS sample buffer (containing DTT) and incubated for 30 min at 50 °C with shaking (700 rpm). 1.111 µL 1 M iodoacetamide (IAA) was added to each sample (final 100 mM IAA), then the samples were mixed and incubated for 30 min at r.t. in the dark. Sample quality was controlled by SDS-PAGE/silver staining using 0.926 µL of each sample (2.5 µg on average for the two lowest temperature samples; 26-well 4–12% Bis-Tris polyacrylamide gels, Novex NuPAGE, Thermo Scientific; 15 min at 50 V, about 1.5 h at 200 V using the recommended MOPS running buffer). For the later MS analysis, 4.63 µL per sample (12.5 µg on average for the two lowest temperature samples) were subjected to short SDS-PAGE (30 min at 50 V) using the same SDS-PAGE setup. The remaining halves of the samples (5.555 µL) were stored as backup samples at −80 °C. The gels containing the MS samples were incubated for 1 h in fixing solution (40% ethanol, 2% acetic acid), stained for 3 min in CBB staining solution (25 mL 0.1% *w*/*v* Brilliant blue G, 0.29 M H_3_PO_4_, 16% saturated (NH_4_)_2_SO_4_ mixed with 6.25 mL ethanol per gel), decolorized with decolorizing solution (25% ethanol, 5% acetic acid) until the gel background was almost colorless and washed with ddH2O until the water remained colorless. The short gel lanes were cut out of the gel on a clean glass plate with a scalpel and each transferred into 0.5 mL LoBind Eppendorf tubes, which were stored at −80 °C.

*In-gel digestion:*

The gel lanes were decolorized and dehydrated by incubating each gel lane twice with 300 µL 40% (*v*/*v*) ethanol in 5 mM tetraethylammonium bicarbonate (TEAB) for 45 min at 55 °C and, thereafter, twice with 300 µL ethanol (p.a., undenatured) for 5 min at r.t., discarding the supernatant after each incubation. The dehydrated gel lanes were then completely dried using vacuum centrifugation (SpeedVac concentrator) and stored at −80 °C. 24 µL 1 µg/µL Trypsin/Lys-C mix (Mass Spec Grade, Promega, V5073, reconstituted in the supplied “Resuspension Buffer”: 50 mM acetic acid, pH < 3) was mixed with 1176 µL 5 mM TEAB in HPLC-MS grade water and 14 µL of the resulting 0.02 µg/µL Trypsin/Lys-C mix solution was added to each dry gel lane. After at least 15 min incubation at r.t. to completely absorb the solution, each gel lane was completely immersed by addition of 60 µL of 5 mM TEAB, followed by a 3.5 h incubation at 37 °C within an incubator. The supernatants of the gel lanes were each transferred into new 0.5 mL LoBind Eppendorf tubes and stored at −80 °C. 21 µL 0.02 µg/µL Trypsin/Lys-C mix (prepared analogously as described above) was added to the gel lanes, respectively, followed by an overnight incubation at 37 °C within an incubator.

*Extraction of the peptides from the gel lanes after digestion:*

The supernatants of the gel lanes after overnight digestion were combined with the first supernatants (after 3.5 h digestion) in the corresponding 0.5 mL LoBind Eppendorf tubes. 15 µL 5% formic acid (FA; in HPLC-MS grade water) was added to each the gel lane. Then, 60 µL 1% FA was added to each gel lane, followed by an incubation for at least 30 min at r.t. The supernatants of the gel lanes were combined with the corresponding previous supernatants. The addition to the gel lanes, incubation, and combining with the corresponding previous supernatants was repeated using again 60 µL 1% FA, then 60 µL 60% ACN in 1% FA, and finally 90 µL of ACN (incubation > 15 min instead of >30 min for the last step). The extracted gel lanes were discarded. Forty percent, respectively, of the collected supernatants containing the extracted peptides were transferred into new LoBind 0.5 mL Eppendorf tubes, shock-frozen in liquid nitrogen, and evaporated to dryness using vacuum centrifugation (SpeedVac concentrator). The remaining 60% were stored at −80 °C as backup.

*TMT labeling:*

The dried peptides were reconstituted each in 5 µL 180 mM TEAB in 10% (*v*/*v*) ACN (repeatedly vortexed and spun down) and allowed to equilibrate to r.t. The tandem mass tag (TMT) labeling reagents (Tandem Mass Tag 10-plex isobaric label reagent set, Thermo Scientific, 0.8 mg per tag; at r.t.) were reconstituted in 41 µL ACN (anhydrous HPLC-MS grade) each, and 2 µL each were added to the reconstituted peptides according to the following scheme and were homogenized immediately by aspiration and dispensing:
TMT label:131130C130N129C129N128C128N127C127N126Treatment temp./°C:36.541.244.047.149.853.356.059.264.067.0

After vortexing and spinning down, the samples were incubated for 1 h at r.t. and, then, quenched by addition of 0.8 µL 5% hydroxylamine each (homogenized immediately after addition) and incubation for more than 15 min at r.t. All samples of a sample set comprising the different TMT labels were combined in the tube containing the sample corresponding to the 67 °C treatment (TMT label 126). The empty tubes of the respective sample set were rinsed successively (TMT label 131 towards TMT labels 130C, 130N, 129C, etc.) using 5 µL 180 mM TEAB in 10% (*v*/*v*) ACN, which was finally combined with the respective combined labeling solutions. The combined TMT-labeled peptide samples of each sample set were shock-frozen in liquid nitrogen, evaporated to dryness using vacuum centrifugation (SpeedVac concentrator), and stored at −80 °C.

*High pH reversed-phase peptide fractionation:*

Offline peptide pre-fractionation was performed using a commercially available kit (Pierce High pH Reversed-Phase Peptide Fractionation Kit, Thermo scientific) according to the manufacturer’s specifications for TMT-labeled peptides. The resulting eight fractions (300 µL each) per sample set were stored at −80 °C.

*LC-MS*/*MS analysis:*

28.4 µL per fraction was evaporated to dryness using vacuum centrifugation (SpeedVac concentrator), reconstituted in 17 µL 0.1% trifluoroacetic acid (TFA), and 15 µL of each resulting sample was subjected to MS measurement, employing the following setup and conditions.

Peptide separation was performed on a Rapid Separation Liquid Chromatography System (Ultimate 3000, Thermo Fisher). First, peptides were concentrated on a trap column (Acclaim PepMap100, 3 µm C18 particle size, 100 Å pore size, 75 µm inner diameter, 2 cm length, Thermo Fisher Scientific, Dreieich, Germany) using a flow rate of 6 µL/min for 10 min with 0.1% TFA as mobile phase. Then, the peptides were separated on the analytical column (Acclaim PepMapRSLC, 2 µm C18 particle size, 100 Å pore size, 75 µm inner diameter, 25 cm length, Thermo Fisher Scientific, Dreieich, Germany) heated to 60 °C using a flow rate of 300 nl/min and a 2 h gradient from 4 to 40% solvent B (solvent A: 0.1% (*v*/*v*) formic acid in water; solvent B: 0.1% (*v*/*v*) formic acid, 84% (*v*/*v*) ACN in water).

Peptides were processed via a nano-source ESI interface equipped with distally coated SilicaTip emitters (New Objective, Woburn, MA, USA) into the online-coupled Orbitrap Fusion Lumos Tribrid mass spectrometer (Thermo Fisher Scientific, Dreieich, Germany; Software: Xcalibur 4.3.73.11, SII 1.5.0.10747, Orbitrap Fusion Lumos 3.1.2412.25), which was operated in positive mode with a spray voltage of 1400 V, an ion transfer tube temperature of 300 °C, and advanced peak determination disabled. Precursor mass spectra were recorded in the orbitrap analyzer within a mass range of 375–1500 m/z and a resolution of 120,000 (maximum injection time 50 ms, automatic gain control target value 400,000, profile mode). For a maximum of three seconds, precursors with charge states +2 to +7 and a minimum intensity of 5000 were isolated with a 0.7 m/z isolation window and were fragmented via collisional-induced dissociation with a normalized collision energy of 35% and 10 ms activation time (Q = 0.25). Precursors that were chosen for fragmentation were excluded from further isolation for the next 60 s. MS2 spectra were recorded with scan rate ‘turbo’ (125,000 Da/s) in the linear ion trap in centroid mode with a maximal injection time of 50 ms and a target value for the automatic gain control set to 10,000. Precursors for MS3 spectra were selected in a mass range of 400–1200 m/z with a 2 m/z isolation window using isobaric-tag-exclusion filtering, with TMT as the reagent tag type, as well as synchronous precursor selection (SPS, maximum 10 notches). The precursors were fragmented via higher-energy collisional dissociation with a normalized collision energy of 65%, and MS3 spectra were recorded in the Orbitrap analyzer within a mass range of 100–500 m/z and a resolution of 50,000 (maximum injection time 105 ms, automatic gain control target value 100,000, centroid mode). The desired minimum points across the peak was set to six for all MSn (n = 1–3) levels.

##### TPP-CCR

*Single-pot, solid-phase-enhanced sample preparation (SP3) modified according to* [39]*:*

For each sample set comprising ten compound concentration range samples, a same volume of the cell extracts containing the fraction of soluble, non-denatured proteins, corresponding to an average of 5 µg total protein, was used for further analysis. The calculated volume was respectively transferred into PCR tubes of a tube strip and supplemented with SDS sample buffer without DTT and bromophenol blue (final concentrations: 7.5% glycerol, 3% SDS, 37.5 mM Tris/HCl pH 7.0) and ddH2O to a final volume of 10 µL, respectively. Reduction and alkylation were performed by addition of 2.5 µL 100 mM dithiothreitol (DTT; incubation: 20 min, 56 °C, 1000 rpm in an Eppendorf ThermoMixer C, covering the tubes with aluminum foil to minimize condensation in the lids) and 3.33 µL 300 mM iodoacetamide (IAA; incubation: 15 min, r.t., in the dark without shaking), as well as 2.5 µL 100 mM DTT (incubation: 15 min, r.t., without shaking; quenching of excess of IAA). Proteins were precipitated on the solid phase (magnetic beads) by addition of 2.5 µL 20 mg/mL bead mix in ddH2O (pre-washed with ddH2O, 1:1 mix from Sera-Mag SpeedBeads cat. no. 45152105050250 and 65152105050250, GE Healthcare), mixing, and the addition of 20.84 µL absolute ethanol (incubation: 15 min, 24 °C, 1000 rpm in an Eppendorf ThermoMixer C). The beads were collected and washed (3× 200 µL 80% ethanol p.a. in HPLC-MS grade water, 1 × 200 µL acetonitrile (ACN; HPLC gradient grade)) using a device for handling of magnetic particles [patent US20100200405A1]. The ACN of the last washing step was completely removed by aspiration without letting the beads dry out.

Tryptic digest was performed on-bead by resuspending (use of an ultrasound bath may be required) the beads in 20 µL 50 mM TEAB in HPLC-MS grade water containing 0.1 µg Trypsin/Lys-C mix and 16.5 h incubation at 37 °C and 1000 rpm (Eppendorf ThermoMixer C, covering the tubes with aluminum foil to minimize condensation in the lids). Tryptic fragmentation of proteins was optimized by addition of another 0.1 µg of Trypsin/Lys-C mix in 2 µL 50 mM TEAB and 4 h incubation at 37 °C and 1000 rpm. After centrifugation for about 1 min in a table top centrifuge and magnetic concentration and retention of the beads at the inner tube wall, the supernatants containing the peptides were transferred into new PCR tubes (of tube strips) and were stored at −80 °C. The beads were discarded.

*TMT labeling:*

Half of the peptide samples (11 µL containing about 2.5 µg peptides) were transferred into new PCR tubes (of tube strips) and allowed to equilibrate to r.t. TMT labeling reagents were reconstituted as described for the TPP-TR samples, and 1 µL each was added to the reconstituted peptides according to the following scheme and homogenized immediately by aspiration and dispensing:
TMT label:131130C130N129C129N128C128N127C127N126Inhibitor conc./µM:2051.250.3130.07810.01950.004880.001220.0003050 *(* vehicle control)

Incubation, quenching (0.8 µL 2.5% hydroxylamine), combining (into the sample corresponding to the vehicle control (TMT label 126)), and successive rinsing (from the lowest to highest temperature, i.e., starting at TMT label 131, using 20 µL 20% (*v*/*v*) ACN), as well as evaporation and storage at −80 °C, was performed analogously to the TPP-TR samples described above.

*High pH reversed-phase peptide fractionation:*

Offline peptide pre-fractionation was performed as described for the TPP-TR samples.

*Mass spectrometric (MS) measurement:*

28.4 µL per fraction was evaporated to dryness using vacuum centrifugation (SpeedVac concentrator), reconstituted in 17 µL 0.1% trifluoroacetic acid (TFA), and 15 µL of each resulting sample was subjected to MS measurement, employing the following setup and conditions.

Peptide separation was performed as described above for the analysis of TPP-TR samples. Peptides were processed via a nano-source ESI interface equipped with distally coated SilicaTip emitters (New Objective, Woburn, MA, USA) into the online-coupled Q Exactive plus hybrid quadrupole Orbitrap mass spectrometer (Thermo Fisher Scientific, Dreieich, Germany; Software: Xcalibur 4.1.31.9, SII 1.4.0.106, Q Exactive Plus MS 2.9-290204/2.9.3.2948), which was operated in positive mode with a spray voltage of 1500 V and the capillary temperature set to 250 °C. For analysis using a data-dependent top ten method, precursor mass spectra were recorded in the Orbitrap analyzer within a mass range of 350–2000 m/z and a resolution of 70,000 (maximum injection time 250 ms, automatic gain control target value 3,000,000, profile mode). A maximum of ten precursors with charge states +2 to +6 were selected by the quadrupole (1 m/z isolation window, 1700 intensity threshold, minimum automatic gain control target 170), fragmented by higher-energy collisional dissociation (normalized collision energy 33), and fragments were then analyzed (profile mode, scan range 200–2000 m/z, resolution 35,000, target for automatic gain control 200,000, maximum injection time 100 ms). Selected precursors were excluded from further fragmentation for the next 40 s.

### 4.3. Data Analysis

#### 4.3.1. Immunoblotting

##### CETSA

In the present immunoblotting melting curve data, the integrated ECL signal intensity (I/a.u.) as a function of temperature (T/°C) followed a sigmoidal trend, which was fitted with the following equation, derived from chemical denaturation theory [64] using R version 4.0.3 (www.r-project.org accessed in October 2020) on a x86_64-w64-mingw32/x64 platform analogously as described [16]:

*I*(*T*) = *I_max_*/{1 + exp[(*T_m_*/*T* − 1)*s*T_m_]},

derived from

*I*(*T*) = *I_min_* + (*I_max_* − *I_min_*)/{1 + exp[−(*a*/*T* − *b*)]}

with *I_min_* = 0 (the lowest integrated ECL signal intensity values always were zero, so there was no plateau), the maximum intensity (top asymptote) fitting parameter *I_max_*, the melting point fitting parameter *T_m_* (= s*a*/*b*), and a slope fitting parameter *s* (= −*b*^2^/*a*).

Separately for each protein/proteoform (MAPK14, MAPKAPK2, MAPKAPK2p, MAPKAPK3, GSK-3a), global fits were performed using the nls() function in R, allowing for different fitted *I_max_* values of the replicates (constant for the different inhibitors, i.e., assuming the absence of an inhibitor effect for the lowest treatment temperatures) and different fitted *T_m_* and *s* values for the different inhibitors (constant for all replicates). The intensity values for each set of melting curves (different inhibitors, same protein, and replicate) were then scaled such that the fitting parameter for the maximal intensity (*I_max_*) was 100%. Fitting and scaling were repeated to self-consistency. Since MAPKAPK2 and MAPKAPK2p showed clearly different initial (lowest temperature points) signal intensities in living cells treated with different inhibitors, the scaling of intensity values was performed individually for each inhibitor (results shown in Figure 1) or only based on AMG548 treatment for MAPKAPK2 (results shown in Supplementary Figure S2). Thermal shifts induced by the inhibitors are reported as Δ*T_m_* to DMSO as the vehicle control in Table 1.

To assess whether inhibitor treatment caused a significant change in melting behavior, the null model, comprising a fit of all scaled replicates of two states (e.g., with and without inhibitor treatment) using one common melting curve (melting point, *T_m_*, and slope, *s*, as fitting parameters; maximum intensity *I_max_* = 100%), was compared to the alternative model, comprising a fit using two melting curves with two (different) melting point and slope parameters, respectively, which was similar to the NPARC method [65]. The comparison was performed using the analysis of variance function in R (anova()).

##### ITDR-CETSA

The integrated immunoblot ECL signal intensities for MAPK14, MAPKAPK2, MAPKAPK2p, and MAPKAPK3 were normalized using the respective GSK-3α intensities in the same lane. The resulting relative intensity (*I*/a.u.), as a function of compound concentration on the molar log_10_ scale (log_10_(c/M)), followed—in the case that an intensity change was observed, i.e., mostly for AMG548 and SB-203580 treatment—a sigmoidal trend (dose–response), which was fitted with the following form of the Hill equation [66] using R (v4.0.3) analogously as described [16].

*I*(log_10_(*c*)) = *I_min_* + (*I_max_* − *I_min_*)/{1 + 10^[*H*(-pEC50 − log^10^(*c*))]^},

where the fitting parameters *I_min_* and *I_max_* denote the minimal and maximal intensity (bottom and top asymptotes of the fitting function), respectively, the fitting parameter pEC50 represents the negative logarithm to base 10 of the half-maximal effective concentration, and the fitting parameter *H* is the Hill coefficient.

Separately for each protein/proteoform (MAPK14, MAPKAPK2, MAPKAPK2p, MAPKAPK3), global fits were performed using the nls() function in R, allowing for different fitted *I_min_* and *I_max_* values for the replicates (constant for the different inhibitors) and different fitted EC50 and *H* values for the different inhibitors (constant for all replicates). The intensity values for each set of dose–responses (different inhibitors, same protein and replicate) were then scaled towards *I_min_* = 0% and *I_max_* = 100%. Fitting and scaling were repeated to self-consistency.

#### 4.3.2. MS Data

*MS data analysis, protein identification, and quantification:*

MS data was processed with MaxQuant (Max Planck Institute for Biochemistry, Planegg, Germany) version 1.6.6.0 for TPP-TR and version 1.6.17.0 for TPP-CCR. If not stated otherwise, standard parameters were used for protein identification and quantification. Searches were carried out based on 74416 Homo sapiens protein entries downloaded from the UniProtKB on 19 June 2019 for TPP-TR or 75777 Homo sapiens protein entries, which were downloaded from the UniProtKB on 27 January 2021 for TPP-CCR using tryptic cleavage specificity (behind K and R) and a maximum of three missed cleavage sites. Methionine oxidation and N-terminal acetylation as well as a carbamidomethylation at cysteine residues were considered as variable and fixed modifications, respectively. First, an initial search was carried out using a precursor mass tolerance of 20 ppm and after recalibration, a second search was performed with 4.5 ppm precursor mass tolerance. Tolerances for fragment spectra were 20 ppm. Peptides and proteins were identified with an FDR of 1%. The eight MS raw files corresponding to the eight high-pH fractions of each sample set were defined as fractions (Fraction 1–8) of the respective experiment in the MaxQuant GUI. The settings “Label-free quantification” and “Match between runs” were disabled. Reporter ion MS3 (TPP-TR) or MS2 (TPP-CCR) with 10plex TMT and a reporter mass tolerance of 0.003 Da were chosen without correction factors for the single TMT labels, because their effects had been found to be negligible in previous tests on published data sets (Pride ID: PXD013774 [18]). Unmodified counterpart peptides were discarded, and advanced ratio estimation was enabled.

##### TPP-TR

In total, 3639 protein groups (without reverse hits, contaminants and “Only identified by site” hits) were identified for the four sample sets (single treatment with AMG-548, SB203580, ERK 11e, or vehicle control) and quantified by their reporter intensities (10 TMT labels coding for ten temperature treatment points) gathered in the proteinGroups.txt file by the MaxQuant software. Separately for each inhibitor treatment and after normalization to the maximum intensity of the ten temperature treatments per protein, melting curve analysis was performed similarly as described for CETSA using the function

*I*(*T*) = *Imin* + (*Imax* − *Imin*)/{1 + exp[(*Tm*/*T* − 1)*s*Tm]},

with *I_min_*, *I_max_*, *T_m_*, and *s* as fitting parameters within the nlsLM() function in R (v4.0.3). This four-parameter fit has been reported [67] to be superior to the three-parameter fit solely used in the TPP-TR package [18,65]. For each protein, the intensities of the ten temperature treatments were normalized using the corresponding fitted *I_max_* values. For some proteins, the data points corresponding to 49.8 °C for ERK 11e treatment and vehicle control appeared as outliers and were excluded from data analysis.

##### TPP-CCR

Using the reporter intensities given in the MaxQuant proteinGroups.txt output, each sample set, i.e., each replicate for each inhibitor consisting of ten inhibitor treatment concentrations, was linearly normalized towards a zero median of the log2 fold changes over all proteins with valid values, median(log_2_FCs), with respect to the sample with the highest intensities within the set, i.e., the sample for which median(log_2_FCs) > 0 with respect to each of the other nine samples. For each protein within that set, the ten reporter intensity values, corresponding to the ten inhibitor concentrations, were then scaled to a range between 0 and 1 (by subtracting, respectively, the minimal value followed by dividing by the difference between maximal and minimal value).

The following fitting procedure was performed separately for each inhibitor (AMG-548, SB203580, ERK 11e) using the nlsLM() function in R (v4.0.3) and the same fitting function as described for ITDR-CETSA.

First, for each protein comprising all replicates, two parameter (pEC50 and *H*) fits were performed with fixed *I_min_* = 0 and *I_max_* = 1 using different starting values for pEC50 (10.7, 8.7, 6.7, 4.7) and *H* (−5, −0.5, 0.5, 5) and by choosing the fit with the lowest standard deviation. Second, for each protein, *I_max_* and *I_min_* were fitted separately for each replicate with constant pEC50 and *H* from the first fit and each replicate was scaled to a range between *I_min_* and *I_max_*. Finally, again, for each protein comprising all replicates, two parameter (pEC50 and *H*) fits were performed with fixed *I_min_* = 0 and *I_max_* = 1, using starting values for pEC50 and *H* from the first fit.

In absence of choice between different models [68], the pseudo-coefficient of determination, *pseudo R*^2^ = 1 − *SS_res_*/*SS_tot_*, was used as a goodness-of-fit measure within the same nonlinear model [69] with *SS_res_* as the sum of squared residuals from the regression and *SS_tot_* as the total sum of squares (sum of squared residuals from the mean of all values).

Reverse hits, potential contaminants, “Only identified by site” hits and proteins without valid reporter ion intensities were removed, and the remaining 4255 protein groups were filtered for acceptable stabilizing dose–response behavior separately for each inhibitor (AMG-548, SB203580, ERK 11e), based on the following parameters: (i) *pseudo R*^2^ > 0.5, (ii) 4.7 < pEC50 < 9.7, (iii) 0.2 < *H* < 5, (iv) standard error of *H* < 1, (v) standard error of pEC50 < 1, and (vi) at least two replicates with a full set of ten valid intensity values.

Cluster analysis was performed using the means of the replicates at each compound concentration, restricted to the filtered proteins (13, 8, and 24 proteins for AMG-548, SB203580, and ERK 11e, respectively) using the heatmap() function with hclustmethod = “complete” in R (v4.0.3).

## 5. Conclusions and Outlook

We suggest smarTPCA as a novel method to unveil unknown PPI in living cells that bridges the gap between (i) the cell extract test for distinguishing between primary and secondary small molecule interactors and (ii) TPCA for PPI prediction in the case where the ligand is a cell-permeable small molecule that can be added in controlled doses. The resulting dose–response characteristics for thermal protein (de-)stabilization, when compared between proteins, offer the potential to discriminate between primary and secondary small molecule interactions and to reveal unknown PPI in living cells.

Because two or more direct target proteins can, in principle, have similar binding strengths to the same compound by chance, especially when the compound is a highly promiscuous inhibitor, multiple compounds may be required to differentiate between primary interaction and co-stabilization. It is likely that direct target proteins differ in their specificity profiles (different binding strengths to different compounds), while co-stabilized proteins should closely resemble the specificity profile of the direct target protein with which they interact. From this perspective, smarTPCA is expected to unfold its full strength when using multiple compounds with similar targets, e.g., kinase inhibitor panels.

Besides the classical in vitro methods for determining PPI such as (Co-)IP and pull-down, to our knowledge, nearly all methods for detecting PPI in living cells [70], such as the yeast two-hybrid assay (Y2H), proximity labeling (BioID, APEX, TurboID etc.), protein-fragment complementation assays (PCAs), colocalization of fluorescently labeled proteins, or methods based on (fluorescence) resonance energy transfer ((F)RET), require protein modifications, leading to more artificial systems and a potential bias in PPI, whereas proximity ligation assays (PLAs) require cell permeabilization and—as antibody-based approaches—prior knowledge of the interaction partners.

Based on our findings, we envision smartTPCA—besides cross-linking mass spectrometry [71] on living cells—as a method to be used for determining previously unknown PPI in living, nonpermeabilized cells that does not require protein modifications.

## Figures and Tables

**Table 1:** Melting curve data of the CETSA experiments shown in Figure 1 (n: number of replicates). Thermal shifts induced by the inhibitors are reported as Δ*T_m_* to DMSO as the vehicle control.

		LIVING CELLS					Cell Extract *^a^*				Δ*T_m_* to DMSO in Cell Extract Compared to Living Cells/°C
Protein	Inhibitor	n	T*_m_*/°C	Std. Error	Δ*T_m_* to DMSO/°C	*p*-Value (ANOVA, to DMSO)	T*_m_*/°C	Std. Error	Δ*T_m_* to DMSO/°C	*p*-Value (ANOVA, to DMSO)	
MAPK14	AMG-548	7	60.58	0.52	14.76	4.1 × 10^−46^ ***^,*b*^	59.15	0.57	13.60	5.8 × 10^−22^ ***	−1.16
	SB203580	4	52.86	0.35	7.04	4.3 × 10^−25^ ***	56.86	0.45	11.32	2.7 × 10^−26^ ***	4.28
	ERK 11e	4	45.56	0.43	−0.26	0.17	45.15	0.53	−0.39	0.64	−0.13
	DMSO	4	45.82	0.36			45.54	0.52			
MAPKAPK3	AMG-548	6	54.68	0.36	6.62	3.7 × 10^−15^ ***	49.31	0.36	2.63	9.0 × 10^−7^ ***	−3.99
	SB203580	3	51.56	0.48	3.50	2.2 × 10^−7^ ***	47.42	0.34	0.74	0.21	−2.76
	ERK 11e	3	48.32	0.46	0.26	0.57	46.13	0.39	−0.55	0.24	−0.81
	DMSO	3	48.06	0.40			46.68	0.36			
MAPKAPK2	AMG-548	6	54.39	0.29	5.18	5.2 × 10^−7^ ***	51.06	0.48	2.96	3.5 × 10^−4^ ***	−2.22
	SB203580	3	50.96	0.56	1.75	0.18	49.35	0.46	1.24	0.13	−0.51
	ERK 11e	3	49.19	1.03	−0.02	0.99	48.33	0.49	0.23	0.91	0.25
	DMSO	3	49.20	1.40			48.1	0.48			
MAPKAPK2p	AMG-548		n.d. *^c^*				50.95	0.6	2.82	0.014 *	
	SB203580		n.d. *^c^*				49.03	0.65	0.89	0.63	
	ERK11e	1	47.18	0.90	−1.07	0.13	48.34	0.58	0.21	0.65	1.16
	DMSO	1	48.25	0.49			48.13	0.70			−0.12
GSK-3α	AMG-548	7	53.26	0.25	−0.71	0.24	48.26	0.45	−0.76	0.31	−0.05
	SB203580	4	54.03	0.33	0.05	0.82	49.77	0.44	0.75	0.45	0.70
	ERK 11e	4	54.11	0.30	0.14	0.94	50.25	0.43	1.24	0.11	1.10
	DMSO	4	53.97	0.31			49.02	0.45			

*^a^* n = 3 for incubation temperatures 36.5, 41.2, 44.0, 59.2, 64.0, and 67.0 °C; n = 4 for incubation temperatures 47.1, 49.8, 53.3, and 56.0 °C. *^b^* *p* ≤ 0.05 (*), *p* ≤ 0.001 (***). *^c^* Not determined because MAPKAPK2p was not detected upon treatment of living cells with AMG-548 or SB203580.

**Table 2:** Data of the ITDR-CETSA experiments shown in Figure 2.

Inhibitor	Protein	Living Cells *^a^*		Cell Extract *^a^*	
		pEC50	Std. Error	pEC50	Std. Error
AMG-548	MAPK14	8.93	0.15	7.90	0.05
	MAPKAPK3	8.97	0.16	8.06	0.13
	MAPKAPK2	8.78	0.17	8.03	0.17
	MAPKAPK2p	8.80	0.12	7.78	0.18
SB203580	MAPK14	5.34	0.13	5.85	0.08
	MAPKAPK3	4.98	0.29	n.d.*^b^*	
	MAPKAPK2	5.20	0.21	n.d.*^b^*	
	MAPKAPK2p	6.39	0.22	n.d.*^b^*	

*^a^* n = 4. *^b^* Not determined due to lack of change in signal intensity.

**Table 3:** Data of the TPP-CCR experiments performed using AMG-548 and SB203580 (see Supplementary Table S3 for ERK 11e) for the proteins filtered for acceptable stabilizing dose–response characteristics and ordered by *pseudo R*^2^ (a measure for the goodness-of-fit).

Inhibitor	Protein	pEC50	Std. Error	*pseudo R* ^2^	n *^a^*
AMG-548	MAPK14	8.33	0.04	0.98	5
	MAPKAPK3	8.60	0.05	0.96	5
	MAPKAPK2	8.60	0.05	0.95	5
	MYLK	8.22	0.15	0.77	5
	USP24	8.57	0.13	0.75	5
	STX4	6.88	0.30	0.71	2
	HN1L	6.41	0.17	0.69	5
	MARS	6.68	0.22	0.64	4
	NUDT1	5.64	0.13	0.62	5
	MAPK8	7.91	0.46	0.58	2
	TRAPPC4	6.39	0.21	0.57	5
	MRPS34	7.22	0.44	0.55	2
	MTMR1	6.98	0.39	0.55	2
SB203580	MAPKAPK2	6.15	0.06	0.94	4
	MAPKAPK3	6.05	0.06	0.94	4
	MAPK14	5.71	0.06	0.93	4
	NAPG	8.71	0.44	0.73	2
	WDR46	7.49	0.42	0.61	2
	MAPK8	6.50	0.33	0.58	2
	MYLK	6.02	0.30	0.58	2
	C14orf166	6.39	0.29	0.52	4

*^a^* Of the five or four replicates analyzed for AMG-548 or SB203580 treated living HL-60 cells, respectively, “n” datasets were obtained exhibiting a full set of reporter ion intensities, which were then used for fitting of dose response curves.

## Data Availability

The mass spectrometry proteomics data have been deposited to the ProteomeXchange Consortium via the PRIDE [72] partner repository with the dataset identifier PXD032942. The other data is available upon request.

## Supplementary Materials

The following are available online at https://www.mdpi.com/article/10.3390/ijms23105605/s1.
