# Supplementary material for: Small Molecule Arranged Thermal Proximity Coaggregation (smarTPCA)—A Novel Approach to Characterize Protein–Protein Interactions in Living Cells by Similar Isothermal Dose–Responses
Source: Int J Mol Sci. 2022 May 17;23(10):5605. doi: 10.3390/ijms23105605 (PMC9147192; doi:10.3390/ijms23105605)
Supplement: Supplementary file 1 [file ijms-23-05605-s001.zip › SupplSchemeFiguresTL2022-05-16a.pdf]

Scheme S1

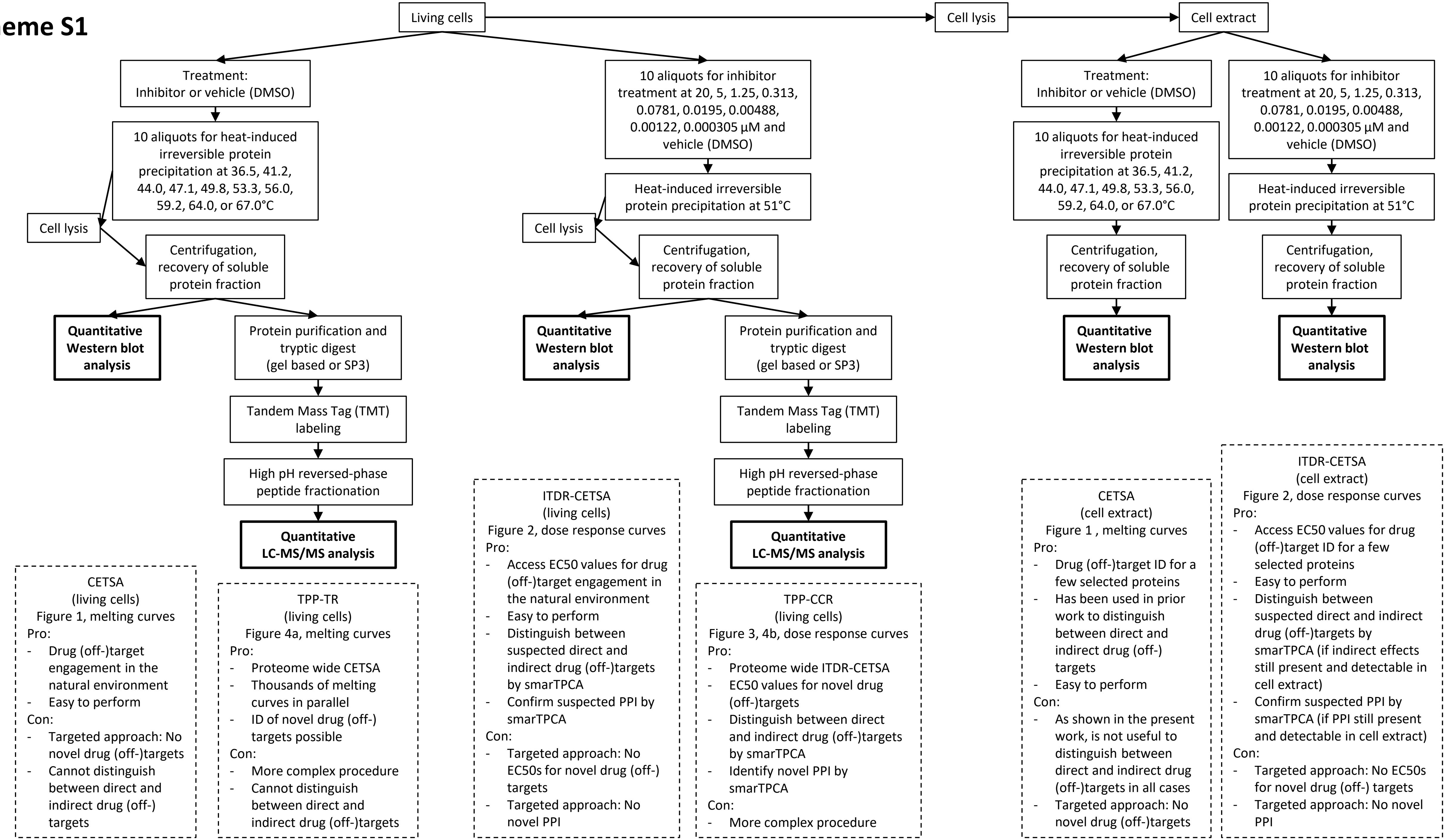
Living cells
Cell lysis
Cell extract
Treatment:
Inhibitor or vehicle (DMSO)
10 aliquots for heat-induced irreversible protein precipitation at 36.5, 41.2, 44.0, 47.1, 49.8, 53.3, 56.0, 59.2, 64.0, or 67.0°C
Cell lysis
Centrifugation, recovery of soluble protein fraction
Quantitative Western blot analysis
Protein purification and tryptic digest (gel based or SP3)
Tandem Mass Tag (TMT) labeling
High pH reversed-phase peptide fractionation
Quantitative LC-MS/MS analysis
CETSA
(living cells)
Figure 1, melting curves
Pro:
- Drug (off-)target engagement in the natural environment
- Easy to perform
Con:
- Targeted approach: No novel drug (off-)targets
- Cannot distinguish between direct and indirect drug (off-) targets
TPP-TR
(living cells)
Figure 4a, melting curves
Pro:
- Proteome wide CETSA
- Thousands of melting curves in parallel
- ID of novel drug (off-) targets possible
Con:
- More complex procedure
- Cannot distinguish between direct and indirect drug (off-)targets
10 aliquots for inhibitor treatment at 20, 5, 1.25, 0.313, 0.0781, 0.0195, 0.00488, 0.00122, 0.000305 µM and vehicle (DMSO)
Heat-induced irreversible protein precipitation at 51°C
Cell lysis
Centrifugation, recovery of soluble protein fraction
Quantitative Western blot analysis
Protein purification and tryptic digest (gel based or SP3)
Tandem Mass Tag (TMT) labeling
High pH reversed-phase peptide fractionation
Quantitative LC-MS/MS analysis
ITDR-CETSA
(living cells)
Figure 2, dose response curves
Pro:
- Access EC50 values for drug (off-)target engagement in the natural environment
- Easy to perform
- Distinguish between suspected direct and indirect drug (off-)targets by smarTPCA
- Confirm suspected PPI by smarTPCA
Con:
- Targeted approach: No EC50s for novel drug (off-) targets
- Targeted approach: No novel PPI
TPP-CCR
(living cells)
Figure 3, 4b, dose response curves
Pro:
- Proteome wide ITDR-CETSA
- EC50 values for novel drug (off-)targets
- Distinguish between direct and indirect drug (off-)targets by smarTPCA
- Identify novel PPI by smarTPCA
Con:
- More complex procedure
Treatment:
Inhibitor or vehicle (DMSO)
10 aliquots for heat-induced irreversible protein precipitation at 36.5, 41.2, 44.0, 47.1, 49.8, 53.3, 56.0, 59.2, 64.0, or 67.0°C
Centrifugation, recovery of soluble protein fraction
Quantitative Western blot analysis
CETSA
(cell extract)
Figure 1 , melting curves
Pro:
- Drug (off-)target ID for a few selected proteins
- Has been used in prior work to distinguish between direct and indirect drug (off-) targets
- Easy to perform
Con:
- As shown in the present work, is not useful to distinguish between direct and indirect drug (off-)targets in all cases
- Targeted approach: No novel drug (off-)targets
10 aliquots for inhibitor treatment at 20, 5, 1.25, 0.313, 0.0781, 0.0195, 0.00488, 0.00122, 0.000305 µM and vehicle (DMSO)
Heat-induced irreversible protein precipitation at 51°C
Centrifugation, recovery of soluble protein fraction
Quantitative Western blot analysis
ITDR-CETSA
(cell extract)
Figure 2, dose response curves
Pro:
- Access EC50 values for drug (off-)target ID for a few selected proteins
- Easy to perform
- Distinguish between suspected direct and indirect drug (off-)targets by smarTPCA (if indirect effects still present and detectable in cell extract)
- Confirm suspected PPI by smarTPCA (if PPI still present and detectable in cell extract)
Con:
- Targeted approach: No EC50s for novel drug (off-) targets
- Targeted approach: No novel PPI

Figure S1

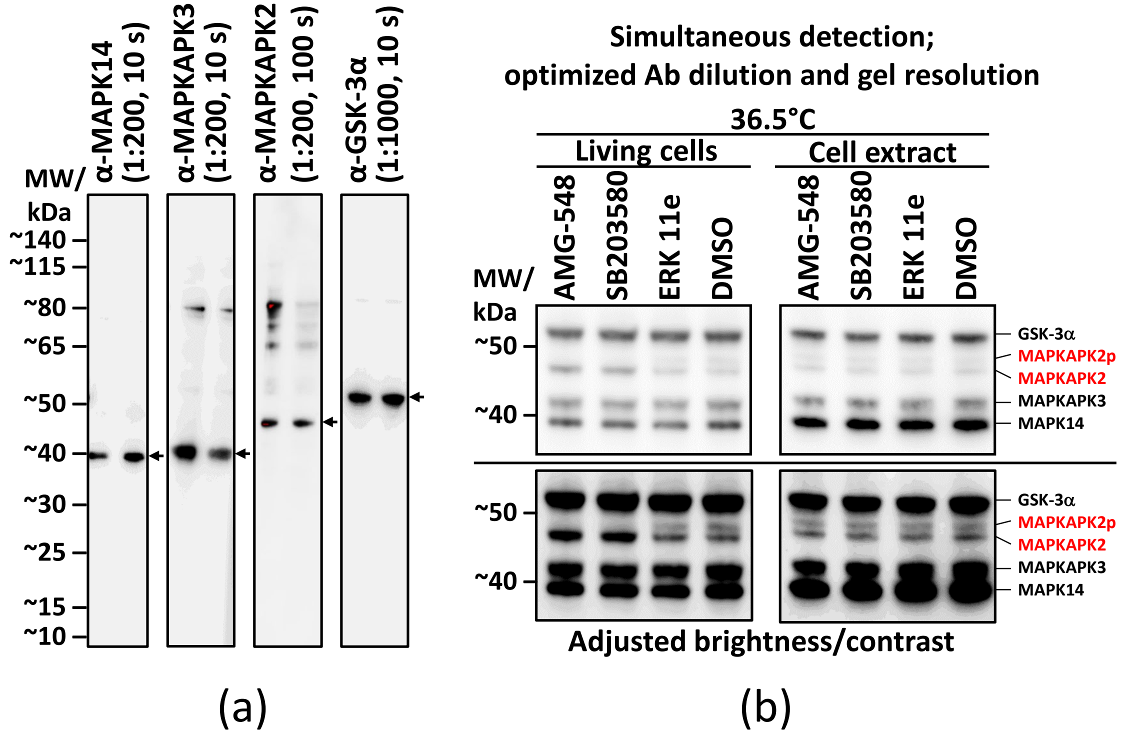

Figure S1. Immunoblot detection of MAPK14, MAPKAPK3, MAPKAPK2, and GSK-3α. (**a**) Separate detection under non-optimized SDS-PAGE and WB conditions (duplicates) from untreated HL-60 cell extract (5 μg total protein per gel lane). The dilution of the primary antibody and the exposure times are denoted in parenthesis above the respective lanes. The specific signals are marked by arrows. No unspecific signals potentially disturbing the simultaneous detection of the proteins from one gel lane are detected below about 60 kDa. (**b**) Simultaneous detection of the proteins from one gel lane under optimized SDS-PAGE (voltage, running time, cooling) and WB conditions (α-MAPK14 (1:2500), α-MAPKAPK3 (1:1300), α-MAPKAPK2 (1:50), and α-GSK-3α (1:1000)). Samples from the lowest CETSA temperature treatment for all inhibitors in living cells and cell extract were analyzed and the resulting immunoblot signals are shown without (top panels) and with adjusted brightness and contrast (bottom panels) for a more detailed presentation of the signals for MAPKAPK2 and MAPKAPK2p.

Figure S2

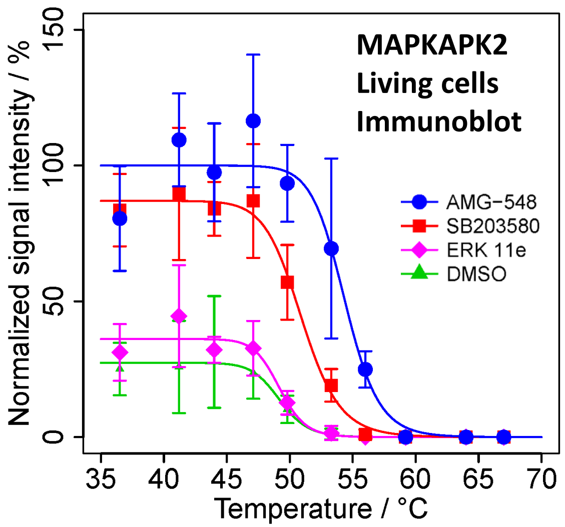

Figure S2. Melting curves of MAPKAPK2 from living cell CETSA experiments without relative (inhibitor treatment) signal normalization.

Figure S3

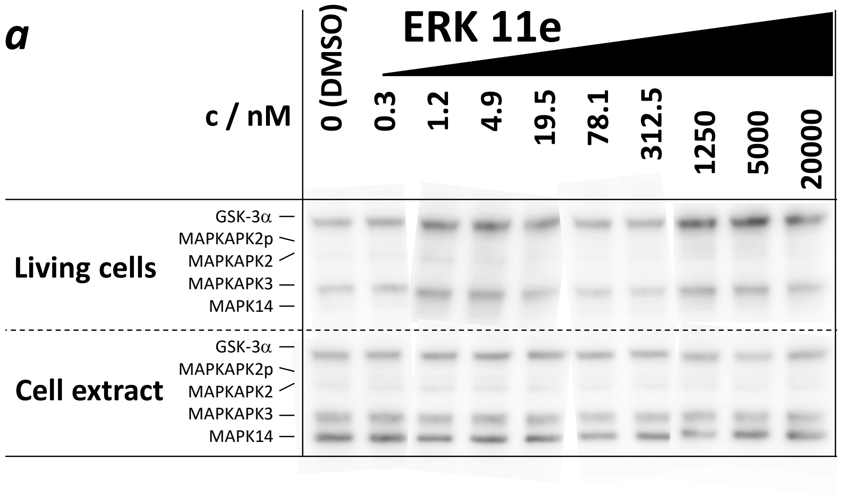

(see Figure S3b for figure caption)

Figure S3

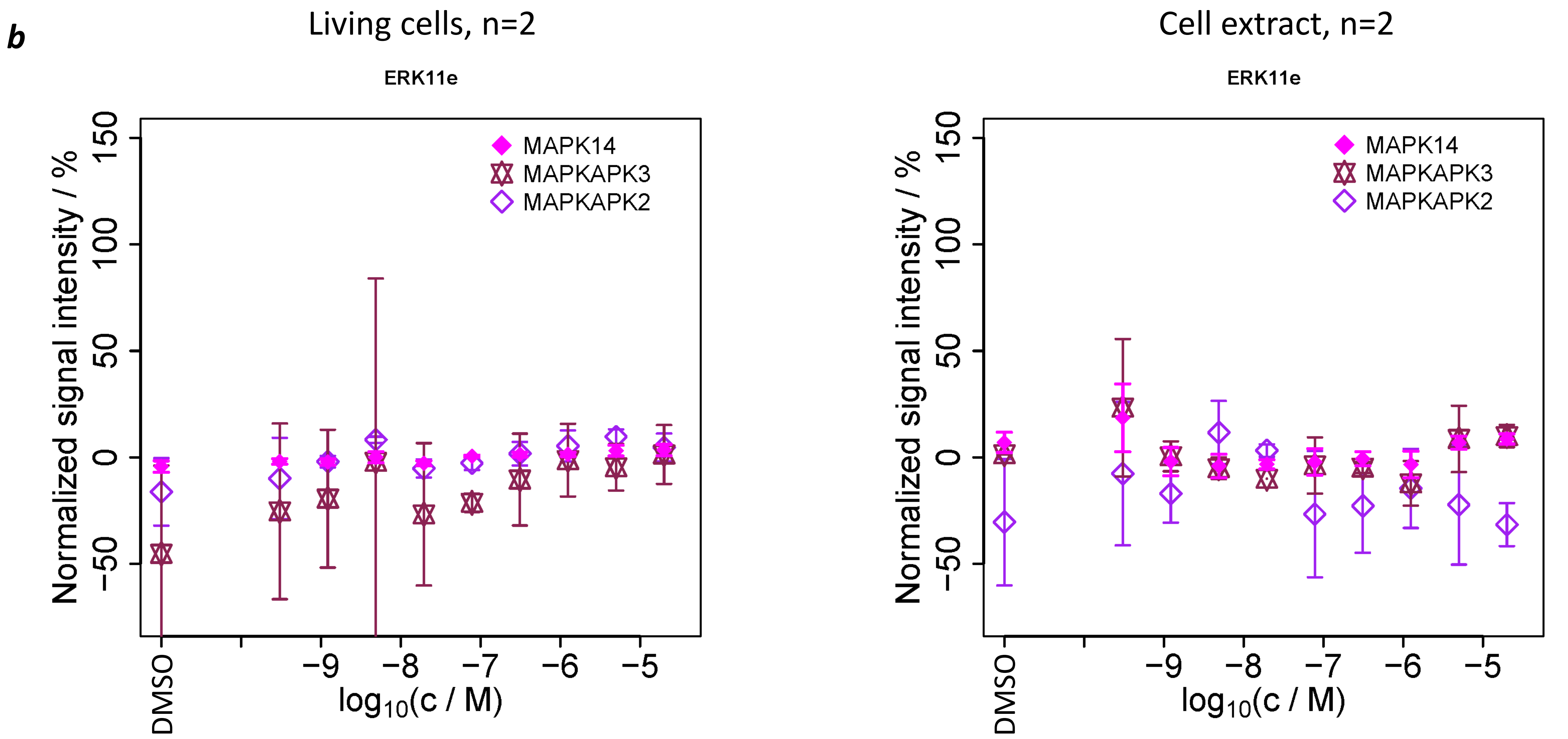

Figure S3. Supplementary data to Figure 2 of the main text showing the absence of dose responses for the control compound ERK 11e in ITDR-CETSA experiments at a constant treatment temperature of 51°C. (**a**) Immunoblots for one of the replicates of ITDR-CETSA experiments for living cells and cell extract, respectively. (**b**) Integrated, normalized signal intensities from the immunoblots. Datapoints and error bars represent means over the replicates +/- one standard deviation. For MAPKAPK2p, no meaningful data could be obtained due to low immunoblot signal intensity.

Figure S4

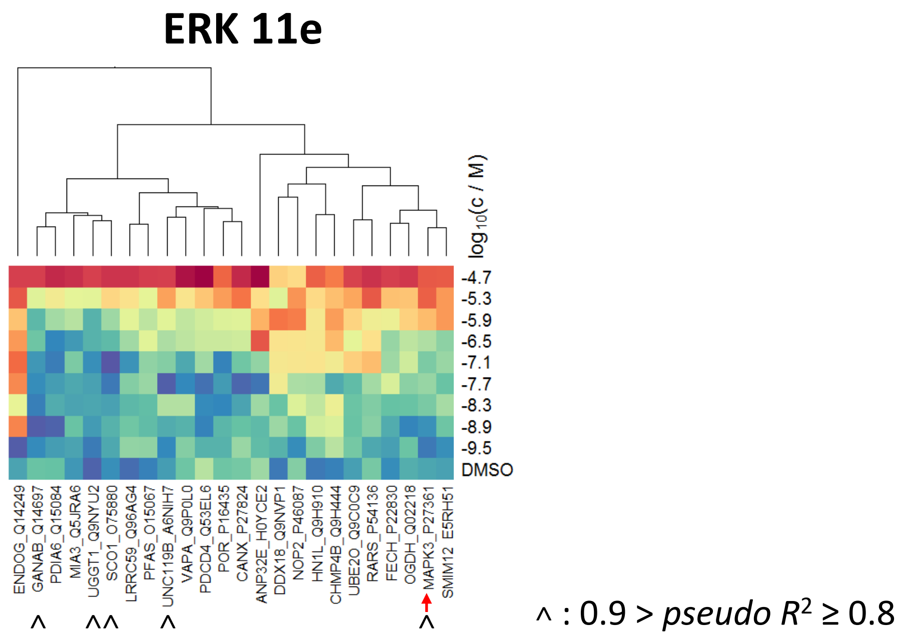

Figure S4. Supplementary data to Figure 3 of the main text showing the heat map after cluster analysis of the filtered (acceptable fit of dose-response curves for stabilized proteins, see experimental section for filter criteria) dose responses (mean over replicates) derived from TPP-CCR experiments in living cells for ERK 11e.

Figure S5

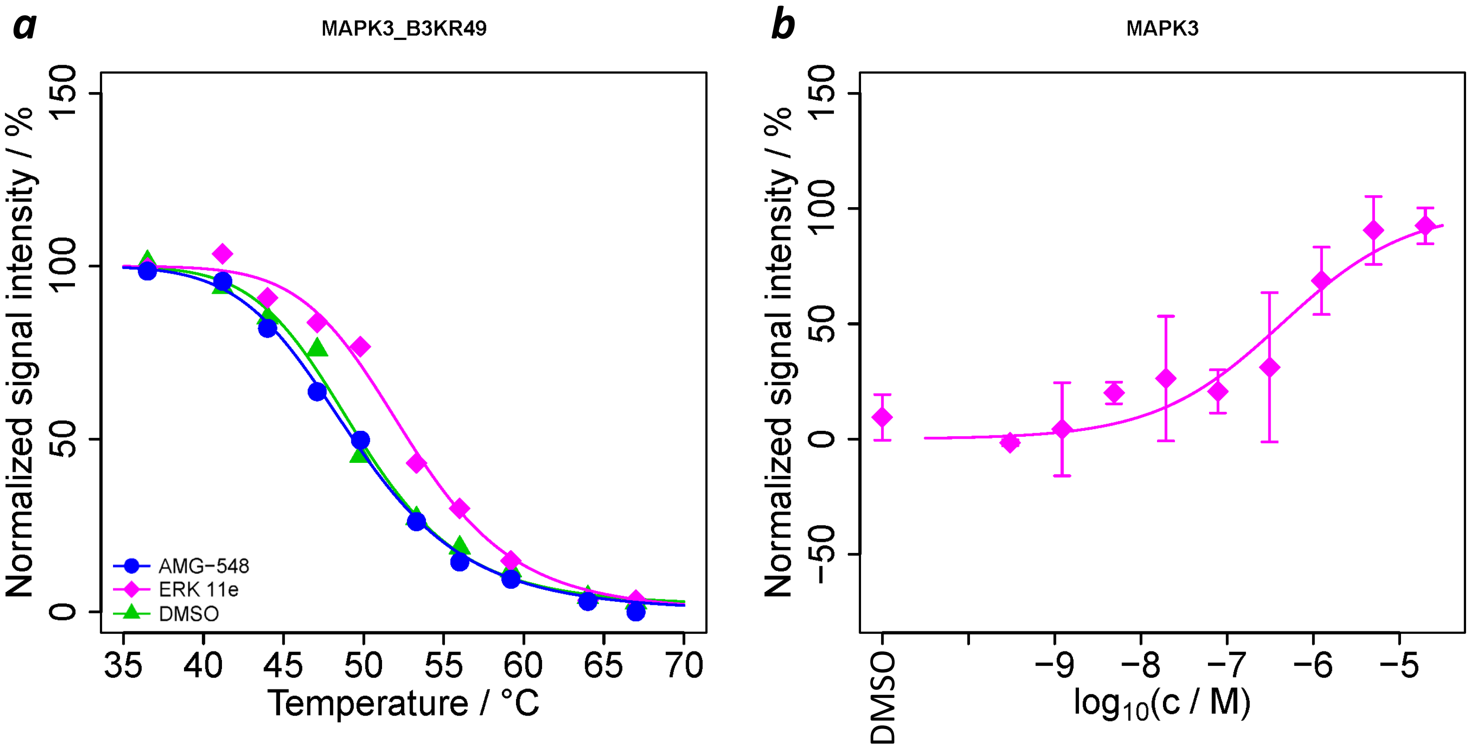

Figure S5. Melting curves (***a***) and dose response curves (***b***) for MAPK3 from the TPP-TR and TPP-CCR experiments. A melting curve for MAPK3 upon SB203580 treatment could not be determined due to missing reporter intensities.

Figure S6

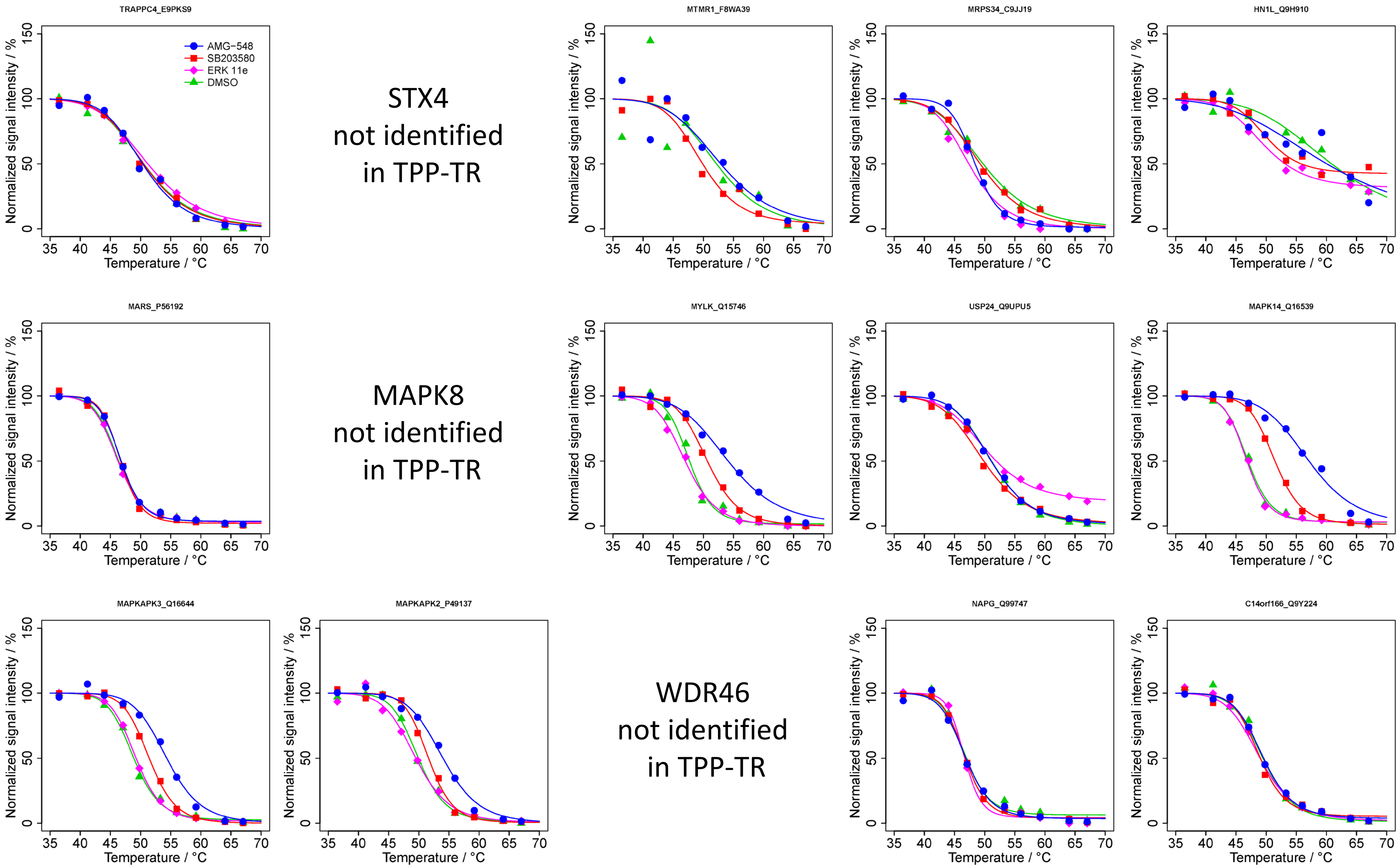

Figure S6: Melting curves from a single set of TPP-TR experiments of the proteins with acceptable stabilizing dose-response behavior in the TPP-CCR experiments shown in Figure 3 of the main text.
